# Quantitative assessment of neonatal health using dried blood spot metabolite profiles and deep learning

**DOI:** 10.1126/scitranslmed.adv4942

**Published:** 2026-01-21

**Authors:** Alan L. Chang, Jonathan D. Reiss, Anthony Culos, Martin Becker, Jonathan A. Mayo, Ivana Marić, Davide De Francesco, Thanaphong Phongpreecha, Camilo A. Espinosa, Samson J. Mataraso, Eloïse Berson, Yeasul Kim, Lei Xue, Feng Xie, Chi-Hung Shu, Ramin Fallahzadeh, Neda H. Bidoki, Maria Xenochristou, Miao Zhang, Jochen Profit, Henry C. Lee, Brice Gaudillière, Martin S. Angst, Steven Hawken, Kumanan Wilson, David K. Stevenson, Gary M. Shaw, Karl G. Sylvester, Nima Aghaeepour

**Affiliations:** 1 Department of Anesthesiology, Perioperative and Pain Medicine, Stanford University School of Medicine, Stanford, CA 94305, USA; 2 Department of Biomedical Data Science, Stanford University, Stanford, CA 94305, USA; 3 Department of Pediatrics, Stanford University School of Medicine, Stanford, CA 94305, USA; 4 Department of Pathology, Stanford University School of Medicine, Stanford, CA 94305, USA; 5 Department of Pediatrics, University of California San Diego, La Jolla, CA 92093, USA; 6 Department of Medicine, University of Ottawa, Ottawa, ON K1H 8M5, Canada; 7 Ottawa Hospital Research Institute, University of Ottawa, Ottawa, ON K1H 8L6, Canada; 8 Bruyère Health Research Institute, Ottawa, ON K1R 6M1, Canada; 9 Department of Surgery, Stanford University School of Medicine, Stanford, CA 94305, USA

## Abstract

Neonatal prematurity leads to considerable morbidity and mortality, partly because of acquired conditions such as bronchopulmonary dysplasia (BPD), intraventricular hemorrhage (IVH), necrotizing enterocolitis (NEC), and retinopathy of prematurity (ROP). Standard gestational age and birthweight-based classifications of prematurity inadequately capture the variation in newborns’ health outcomes, creating an urgent need to develop risk stratification tools for vulnerable newborn infants to initiate the most appropriate care pathways as early as possible. We hypothesized that the metabolic profiles of newborn infants capture additional risk information beyond current measures. A total of 13,536 newborn screening (NBS) blood spot tests from preterm infants in California with linked clinical outcomes of prematurity were used to develop an NBS-based metabolic health index to stratify preterm infants at risk for BPD, IVH, NEC, and ROP (12,096 cases with one or more conditions and 1440 controls) through a deep learning model that provides a single index score in tandem with subgroup discovery to identify individuals with the strongest metabolite biomarker signals for adverse outcomes of prematurity. This metabolic health index captured risk signals that were distinct from gestational age and birthweight and outperformed other machine learning algorithms and clinical risk variable-based models in stratifying at-risk individuals for adverse outcomes of prematurity. The metabolic health index was externally validated in an independent retrospective cohort of 3299 very premature newborns from Ontario, Canada (2117 cases and 1182 controls), which recapitulated common metabolic risk subgroups. In summary, combining widespread metabolite screening with deep learning established a generalizable biological risk metric of prematurity.

## INTRODUCTION

Preterm birth, defined as birth before 37 weeks of gestation, is associated with major health risks (1). Bronchopulmonary dysplasia (BPD), intraventricular hemorrhage (IVH), necrotizing enterocolitis (NEC), and retinopathy of prematurity (ROP) are four grave acquired conditions of prematurity that result in substantial morbidity and mortality (1–5). Gestational age (GA) and birthweight (BW) classifications of preterm birth are of limited value for risk stratification because the health outcomes of infants with similar GA and BW can vary substantially (6). Improved newborn risk assessment is therefore a critical clinical need to enable early stratification to appropriate care pathways, limit the impact of subsequent risk-amplifying exposures for adverse outcomes, and guide the design of future interventions on the basis of biologic metrics (7, 8). One highly promising strategy for developing comprehensive newborn risk assessment is linking biology to risk stratification through routinely collected biological specimens and advanced artificial intelligence approaches. Once identified, these biological risk signatures could then be readily implemented as extensions of preexisting newborn screening (NBS) strategies.

In the United States, United Kingdom, and some European, Asian-Pacific, and Latin American countries, every newborn undergoes NBS via heel- stick dried blood spot after birth to detect severe genetic metabolic disorders (9–12). If a metabolic disorder is detected, then dietary or enzyme-based interventions can be instituted to mitigate disease-associated morbidity from end-organ damage caused by metabolic intermediates. In addition to serving as a readout of metabolic disease, NBS metabolites vary according to the developmental status of the newborn, which implies temporal biologic patterning of metabolic maturity (13, 14). The timing of NBS sample collection (which occurs ideally between 24 and 48 hours after birth) occurs within the window of clinical care exposures and the onset of adverse prematurity-associated outcomes, a timing window that can potentially be adopted for a clinically relevant prognostic test for risk determination. IVH (90%) occurs within 96 hours of delivery, whereas additional adverse outcomes of prematurity typically occur with a latency of several weeks. Newer accepted definitions of BPD are based on an oxygen requirement at 36 weeks postmenstrual age (PMA) (15). Most preterm infants have some degree of ROP when first examined by 31 weeks PMA or 4 weeks postnatally, and ROP treatment is a matter of managing its severity (16). NEC is associated with enteral feedings and gut colonization and typically occurs between 28 and 32 weeks PMA (17). NBS metabolites have previously been shown to reflect prematurity-associated metabolic dysregulation (18, 19). Thus, we hypothesized that distinct adverse outcomes of prematurity share a common underlying biology that can be captured by NBS metabolite profiles and artificial intelligence techniques such as using deep learning models to extract higher-order patterns from data for classification purposes. We used quantitative metabolite results from more than 10,000 routine NBS dried blood spots obtained from preterm live births in California linked to clinical outcomes to develop biological data-driven risk stratification using deep learning to demonstrate that an NBS-derived metabolic health index can predict the likelihood of acquiring one of four adverse neonatal outcomes of prematurity: BPD, IVH, NEC, or ROP.

## RESULTS

### Study population, clinical and outcomes data, and linkage to NBS metabolite profiles

**Fig. 1.**
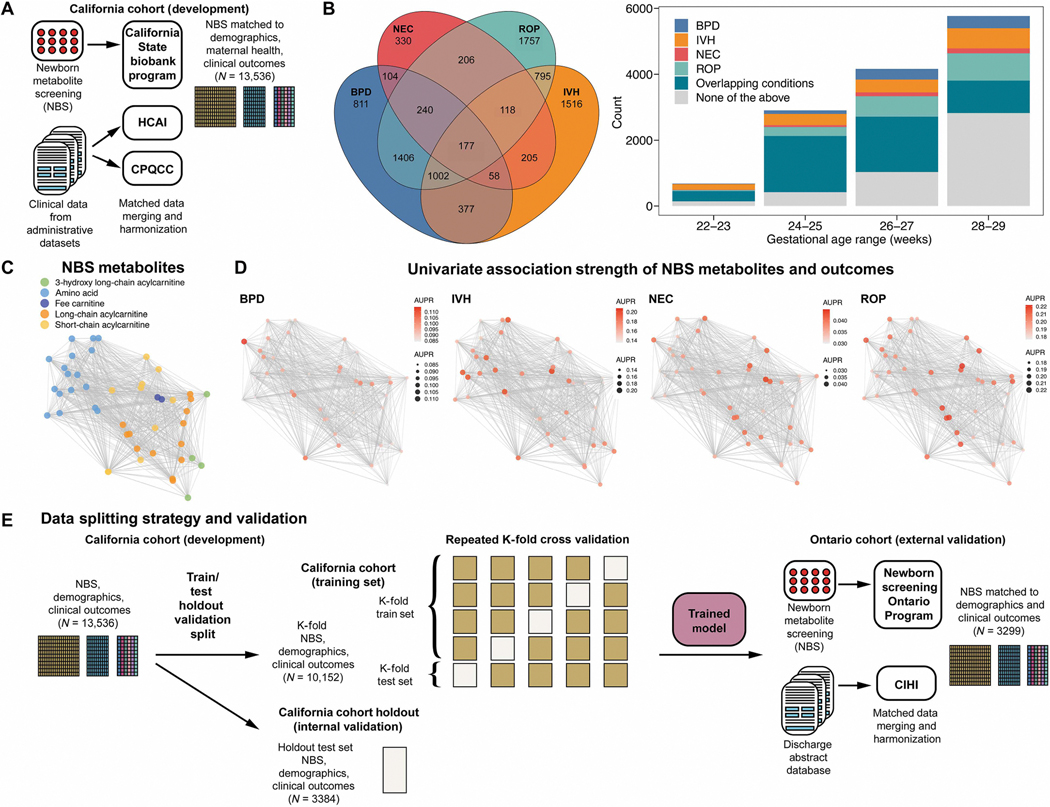
Conceptual framework for metabolic health index–based risk stratification using NBS metabolites. (**A**) Metabolite values obtained from newborn screen results in California were merged with clinical records sourced from HCAI and CPQCC, representing a selection of preterm singleton live births with a gestational age range of 22 to 29 weeks. NBS metabolites, clinical demographics, and newborn outcome information from this cohort were used to train a metabolic health index for better understanding of the health of preterm infants. (**B**) Left: Venn diagram showing numbers of infants with BPD, IVH, NEC, RO P, or more than one condition in preterm infants. Right: Distribution of neonatal conditions across GA age ranges. Overlapping conditions are defined as samples where more than one condition has been indicated in the clinical record, covering the areas of overlap in the Venn diagram. (**C**) Spearman correlation was calculated for all pairs of NBS metabolites embedded using t-distributed stochastic neighbor embedding (t-SNE) and visualized in a graph structure for correlation analysis. Nodes represent individual metabolites, and edges are drawn between statistically significant metabolites subject to Bonferroni correction (two-sided Fisher’s asymptotic *P* < 0.05 used for correlation). Each node is colored by a broader metabolite category. (**D**) Metabolite association with outcomes of prematurity through AUPRC obtained by using metabolite values as outcome thresholds. Size and color of each node correspond to univariate AUPRC. (**E**) The California cohort was used as a development cohort for metabolic health index model development and was further split into a training set for repeated K-fold cross-validation (CV) and a holdout test set for internal validation. An external validation cohort of individuals with a GA of less than 32 weeks was drawn from the Newborn Screening Ontario (NSO) program, with demographic and clinical outcome data drawn from the discharge abstract database through the Canadian Institute for Health Information (CIHI).

Neonates with the diagnoses of BPD, IVH, NEC, or ROP were considered cases and compared with those of similar GA without diagnoses of the above conditions (controls). Two study cohorts were drawn from broader datasets derived from the California State Biobank and Newborn Screening Ontario (NSO) programs, respectively. The California cohort was used for model development, whereas the Ontario cohort was used as an independent external validation cohort. In the California cohort, NBS metabolite measurements and clinical data of 13,536 preterm infants were obtained from the California State Biobank, the Department of Health Care Access and Information (HCAI) [known as the Office of Statewide Health Planning and Development (OSHPD) at the initial data harmonization time point], and the California Perinatal Quality Care Collaborative (CPQCC) (Fig. 1A). The four adverse outcomes of prematurity were confirmed by reviewing the linked clinical data for infants with diagnostic codes reflecting the four morbidities during the newborn hospital stay. The California cohort included 1439 infants with NEC, 5718 with ROP, 4189 with BPD, 4260 with IVH (counts included infants with multiple overlapping conditions), and 1440 infants without any of these conditions (Table 1 and Fig. 1B). The study population was notable given a very low population frequency of infants born at <29 weeks of gestation and a very high associated morbidity rate in this GA range. Because most of these infants had adverse conditions of prematurity, the case-control ratio was higher than that in typical studies closer to the term infant range. Collectively, these data represented a diverse collection of NBS metabolite profiles, clinical outcomes, and demographics in preterm infants ranging from 22 to 29 weeks of GA (encompassing both extremely preterm and very preterm categories by standard definitions). For brevity, we refer to this cohort as “very preterm infants” throughout this manuscript. The cohort was limited to very preterm infants for predictive power considerations and because of the close link between very preterm infant biology and disease etiology for the adverse outcomes in this study.

The relationships between the amino acids, short-chain acylcarnitines, and long-chain acylcarnitines in the NBS panel were determined using correlation network analysis on the California development cohort (Fig. 1C). NBS panel acylcarnitines screen for primary fatty acid oxidation defects and other organic acid handling disorders, whereas additional analyte measurements screen for amino acid metabolism disorders (20). Amino acids were spatially distinct from acylcarnitines in the projected space of the correlation network, whereas long-chain and short-chain acylcarnitines demonstrated more vicinity overlap, with 3-hydroxy long-chain acylcarnitines occupying the periphery. The spatial separation of major NBS metabolite categories implied that broad metabolite types contain distinct biological information (consistent with the inclusion of these specific analytes in the NBS as surrogates of targeted metabolic pathway activity). To understand how individual metabolites relate to neonatal outcomes, univariate area under the precision-recall curve (AUPRC) values for each outcome (including infants with multiple outcomes) were calculated using measurement values for each NBS metabolite (Fig. 1D and table S1). Multiple individual amino acids were associated with IVH (tyrosine, citrulline, methionine, proline, alanine; all empirical *P* < 0.005 by permutation test). In contrast, ROP demonstrated a strong category-level enrichment for short-chain acylcarnitines (Fig. 1E) (Fisher’s exact test, *P* = 0.0292). The analytes C-5 acylcarnitine (C-5) and the analyte ratio of free carnitine (FC) to C-16 acylcarnitine (C-16) and C18:1 acylcarnitine (C18:1) [FC/(C-16 + C-18:1)] were strongly associated with NEC (*P* < 0.005 for both analytes), an observation in line with previous findings (table S1) (21). Additional analysis of directional correlation changes in infants with the designated outcomes revealed pairwise metabolic changes associated with each newborn condition (fig. S1 and table S2). We identified pairs of metabolites that exhibited common core dynamic behaviors across all four adverse outcomes of prematurity [C-12:1 acylcarnitine (C-12:1) and arginine (*P* < 0.005 for all outcomes by Fisher’s Z test for paired correlations), leucine/isoleucine and C-16 (*P* < 0.0001 for all outcomes), leucine/isoleucine and the phenylalanine/tyrosine ratios (*P* < 0.0005 for all outcomes), the phenylalanine/tyrosine ratio and C-5 (*P* < 1 × 10^−8^ for all outcomes), and arginine/ornithine ratio and 5-oxoproline (*P* < 0.0005 for all outcomes)]. In other words, these pairs of metabolites change consistently in the presence of each of the adverse outcomes of prematurity. In contrast, a few pairs demonstrated directional changes that occurred specifically to a single outcome. Among the largest outcome-specific correlation changes in terms of absolute magnitude, we found the most metabolite pairs specifically disrupted in the context of NEC, including the arginine/ornithine ratio and C-14 acylcarnitine (C-14) (*P* = 4.77 × 10^−6^ by Fisher’s Z test for paired correlations), C-12 acylcarnitine (C-12) and arginine (*P* = 1.62 × 10^−10^), C-18:2 acylcarnitine and C-12 acylcarnitine (*P* = 9.15 × 10^−8^), C-8 acylcarnitine to C-10 acylcarnitine ratio (C-8/C-10) and 5-oxoproline (*P* = 2.49 × 10^−5^), and citrulline/arginine ratio and C-12 (*P* = 2.42 × 10^−7^). For BPD, we found that the ornithine and C-14OH acylcarnitine (C-14OH) (*P* = 3.19 × 10^−6^), phenylalanine/tyrosine ratio and phenylalanine (*P* = 1.06 × 10^−10^), and citrulline/arginine ratio and C:14– 1 acylcarnitine (C14:1) (*P* = 7.17 × 10^−5^) ratios were the top disrupted pairs. Valine and C-14OH (*P* = 3.75 × 10^−5^), leucine/alanine ratio and C-18 acylcarnitine (C-18) (*P* = 0.0144), and FC/(C-16 + C-18:1) ratio and ornithine (*P* = 3.83 × 10^−5^) were the metabolite pairs with the largest dynamic changes for IVH.

### Development of a deep metabolic health index for risk stratification in the premature newborn

In the California discovery/development cohort, we used K-fold splitting on the training set while reserving a subset of the data as a holdout test set for internal validation and kept the Ontario cohort strictly sequestered for external validation (Fig. 1E). The Ontario cohort consisted of a very preterm infant cohort of 3299 individuals with NBS metabolite measurements from the NSO program matched to International Classification of Diseases (ICD)–coded outcomes and other clinical information obtained from linkage to the Discharge Abstract Database (DAD), drawn from a larger cohort of live-born individuals developed and maintained by the Canadian Institute for Health Information (CIHI) and the Canadian Institute for Clinical Evaluative Sciences (ICES) (22–24). The Ontario cohort included 303 infants with NEC, 1568 with ROP, 616 with BPD, and 815 with IVH (counts included infants with overlapping conditions) (Table 2). These NSO datasets were linked using unique encoded identifiers and analyzed at ICES.

Because the numbers of individuals in the California development cohort were in the thousands for each outcome, we reasoned that this dataset size would support the development of machine learning approaches for outcome risk stratification. Therefore, we selected deep learning algorithms to derive metabolic risk stratification through an approach well suited for complex biological datasets with multiple outcomes that uses the flexibility of deep learning to derive a single score while leveraging information from multiple outcome labels simultaneously (a training setup known as “multitask learning”) (25). To address the hypothesis that NBS metabolites are informative of a shared vulnerability for multiple outcomes because of common biology, we designed a deep learning architecture that takes NBS metabolite values and uses multilayer perceptron layers as a way to learn nonlinear relationships between metabolites and the four outcomes of BPD, IVH, NEC, and ROP simultaneously. One key advantage of the multitask architecture in deep learning is the ability to account for label overlap and the combinatorial impact of multiple outcomes. These types of models excel at using the joint information from features to identify multiple sample types where such joint information exists. We also limited the width and depth to keep the total parameter count of our deep learning models relatively small to capture complex nonlinear relationships while limiting the potential for overfitting.

**Fig. 2.**
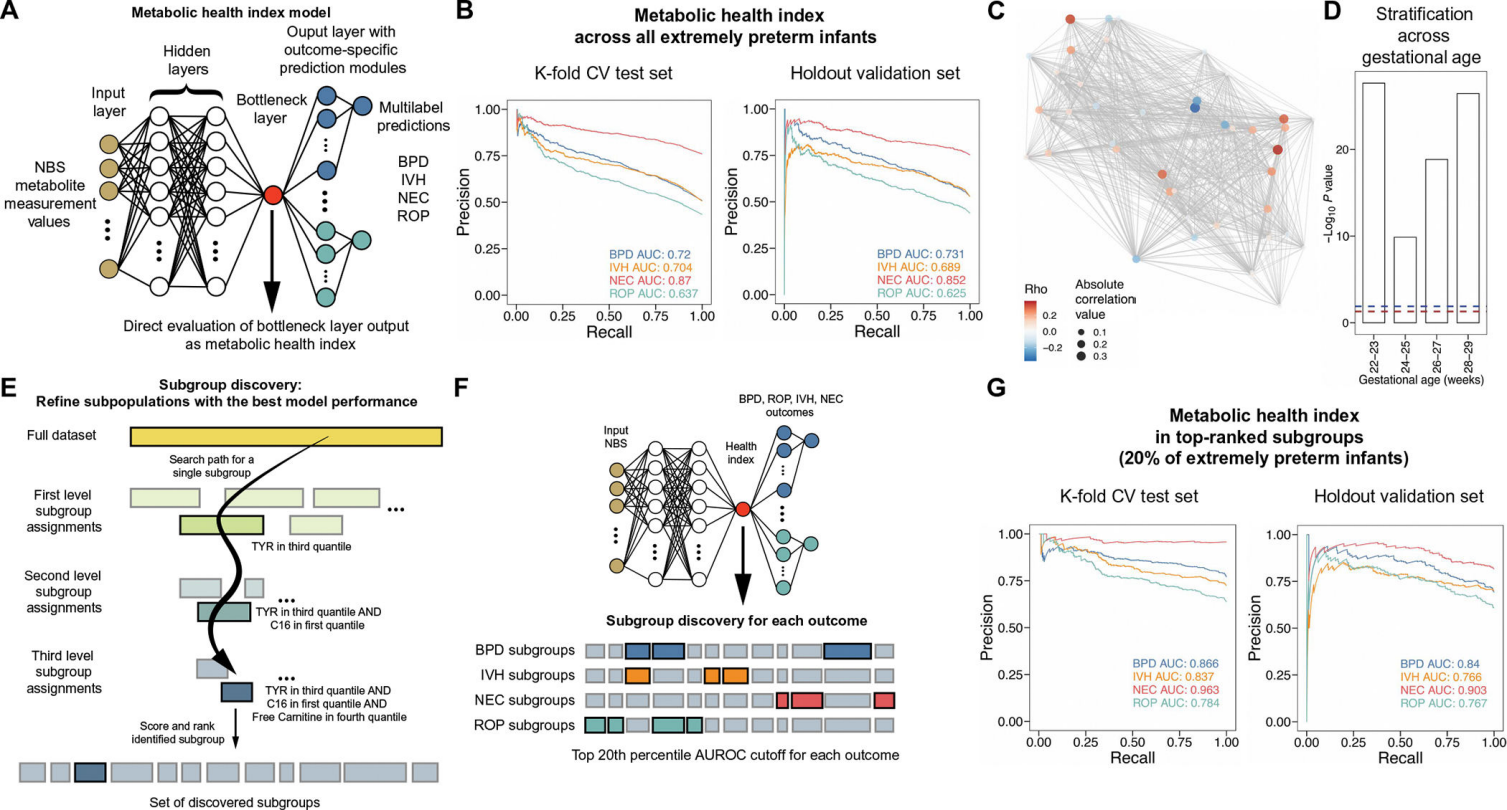
A unifying metabolic health index. (**A**) A bottleneck layer was used to develop a metabolic health index that compresses NBS metabolite profiles into a single risk score that encompasses risk for BPD, IVH, NEC, and ROP. (**B**) Bottleneck output from a model with a single bottleneck unit was evaluated as a single metabolic health index to calculate AUPRC on the K-fold CV test set and the holdout validation set for discerning healthy infants from infants with the four neonatal outcomes. (**C**) Correlation network of metabolites where nodes are colored by Spearman correlation coefficient with the 1-unit bottleneck output. The size of each node is proportional to the absolute value of the Spearman correlation coefficient between metabolite values and 1-unit bottleneck output. (**D**) Infants were separated by GA range, and metabolic health index values were compared between infants with and without any adverse outcomes. −Log_10_ P values for t tests between health index scores for infants with BPD, IVH, NEC, and ROP compared with infants without any of those neonatal outcomes across GA ranges in the study. Dashed horizontal lines correspond to *P* < 0.05 with Bonferroni correction and without Bonferroni correction for multiple comparisons (blue dashed line and red dashed line, respectively). (**E**) Subgroup discovery proceeded iteratively at each level by dividing the dataset into subgroups defined on the basis of quantile cuts of NBS metabolites, illustrated with hypothetical subgroup descriptions. At the end of the procedure, subgroups were scored and ranked on the basis of predefined scoring criteria for further analysis. (**F**) Subgroup discovery was run independently for each of the four adverse outcomes of prematurity using the top 20th percentile AUROC scores as a cutoff for subgroup inclusion. At the end of the subgroup discovery procedures, top subgroups were obtained where the metabolic health index was best able to distinguish between healthy and affected infants for each of the four adverse outcomes of prematurity. (**G**) Subgroup discovery was used to find subpopulations enriched for AUROC performance by the metabolic health index. Precision-recall curves are shown for the K-fold CV test set and holdout validation set as in (B) for the top scoring subgroups comprising 20% of the total dataset.

Initial experiments using deep multitask models powered solely using NBS metabolite values [evaluated using area under the receiver operating curve (AUROC) and AUPRC for BPD, IVH, NEC, and ROP on the K-fold cross-validation test set and holdout validation set] demonstrated that the NBS metabolic profiles of infants with adverse outcomes of prematurity had some risk stratification potential (table S3). We next asked how best to consolidate the risk outcomes and tie them directly to metabolite biomarkers. Deep neural networks can compress biological feature information by drastically reducing the number of hidden units in a middle layer (25). A bottleneck layer was implemented as part of the multitask deep neural network to compress the risk information from all four adverse outcomes into a single score, in effect forcing the network to leverage all of the metabolic risk signals into a scalar value defining a metabolic health index for risk stratification (Fig. 2A). Using this metabolic health index, newborns without the four conditions were effectively separated in both test and internal validation sets (Fig. 2B and table S4). Correlation analysis revealed that the metabolic health index represented a combination of strong metabolic signals, the strongest of which were C-16, the ornithine/citrulline ratio, and C-4 acylcarnitine (C-4), which had Spearman correlation coefficients above 0.3 with the metabolic health index (Fig. 2C and table S5). The difference in metabolic health index scores between infants with and without adverse neonatal health outcomes was significant across all very preterm GA ranges (*P* < 1 × 10^−9^; Fig. 2D). To evaluate NBS metabolite sufficiency for model performance, iterative metabolite removal from the models using AUPRC order identified fundamental groups of metabolites that contained information related to the neonatal outcomes (fig. S2, A to C). Together, these results indicated that a core set of NBS metabolic analytes (C-16, C-18:1, C-18:2, the ornithine/citrulline ratio, C-4, and C-5) formed the basis of a single metabolic health index capable of identifying premature infants who were not at risk for the four studied adverse outcomes of prematurity.

### Subgroup discovery to refine the metabolic health index in heterogeneous populations

The risk profiles of very preterm infants are likely to be heterogeneous in terms of adverse outcomes that cannot be sufficiently distinguished on the basis of clinical features alone. In addition, it is also likely that not all cases with adverse outcomes are associated with specific biological dysfunction reflected in the NBS metabolic analytes because the original design of this metabolic panel was to capture defined inborn errors of metabolism. Thus, for the development of better care management approaches, it may be valuable to understand which individuals can be identified using metabolic risk stratification to deconvolute the recognized heterogeneity of relying on GA and BW alone. Subgroup discovery is a data-mining technique for partitioning subpopulations on the basis of prespecified rules and scoring criteria (Fig. 2E) (26, 27). Using NBS metabolite quantile values to define subgroups and subgroup-specific model AUROC for each of the four outcomes as scoring criteria, we identified outcome-specific subgroups on the basis of the single score metabolic health index model (Fig. 2F). Subgroup discovery yielded patient subsets comprising about 20% of the whole study population where algorithmic stratification using the metabolic health index achieved improved precision and recall (Fig. 2G) (AUPRC—BPD: 0.850, IVH: 0.786, NEC: 0.903, and ROP: 0.767 in a holdout validation set drawn from the California cohort) as well as improved AUROC (tables S3 and S6). The combination of metabolic health index and subgroup discovery methods outperformed elastic net, Lasso, random forest, XGBoost, neural networks alone, and gradient boosting (fig. S3). Furthermore, the top 20th percentile of individuals identified by subgroup discovery was not the equivalent to identifying the 20th percentile of individuals by low BW or GA (fig. S4), although there were statistically significant differences in BW means at the 28- to 29-week GA range (*P* < 0.001). These results suggest that distinct metabolic profiles can identify subpopulations of newborns at heightened risk for adverse outcomes secondary to the biologic metric provided by the NBS.

### Interaction between clinical risk factors and the metabolic health index

The combination of subgroup discovery and the metabolic health index also outperformed linear models that use clinical variables commonly used in neonatal risk calculators such as infant sex, GA, BW, and Apgar scores [a composite test scoring appearance, pulse, grimace (reflexes), activity, and respiration at 1 and 5 min after birth] (table S7) (6, 28). Integrating these clinical risk variables directly into metabolic health index models resulted in improved performance, suggesting that the metabolic health index captures aspects of disease biology distinct from the clinical variables (table S8). As an orthogonal approach to evaluate the potential for integrating clinical variables into the metabolic health index, a series of statistical comparisons was performed for clinical factors that could be related to risk for adverse outcomes in our study population, including maternal race-ethnicity, paternal race-ethnicity, infant sex, and maternal age (table S9). Similar to previous reports, male infant sex was enriched as a risk score for IVH (Bonferroni-adjusted *P* = 0.0478) and ROP (Bonferroni-adjusted *P* = 4.685 × 10^−5^), which was more significant across outcomes than the other demographics and clinical factors (table S9) (29). We trained a separate health index model that uses NBS metabolites and infant sex as features and found strong performance with subgroup discovery refinement in terms of ROC curves (BPD AUROC: 0.91, ROP AUROC: 0.86, IVH AUROC: 0.88, NEC AUROC: 0.89) and precision-recall curves (BPD AUPRC gain: 1.75, ROP AUPRC gain: 1.90, IVH AUPRC gain: 1.73, NEC AUPRC gain: 1.16) (fig. S5A). To interrogate how infant sex is used by this model, feature importance analysis was performed for the health index model and revealed that infant sex did contribute meaningfully to model predictions but did not predominate over other metabolite features in terms of average feature importance (fig. S5B), further supporting the earlier findings that clinical variables provide independent information from the metabolic health index model. We next trained a metabolic health index model that incorporated infant sex, GA, and BW as input features into the full modeling framework. This proof-of-concept demonstration of a combined metabolic-clinical health index further strengthened the risk stratification of BPD, ROP, and NEC (table S10). These results suggest that clinical variables can be directly integrated into the metabolic health index alongside NBS metabolites to create holistic risk stratification that accounts for prior risk associated with clinical factors.

### Dependence of the metabolic health index models on the presence of the four adverse outcomes of prematurity

To assess how critical the shared information of each adverse outcome of prematurity was for the multitask model architecture aspect of the metabolic health index model, a series of ablation experiments was performed where the model was trained for three of the four adverse outcomes of prematurity, iteratively removing a different adverse outcome at a time. Even in these cases, the health index model performed well in the top 20th percentile of subgroups after subgroup discovery (fig. S6, A to D). The model without IVH (fig. S6A) was a particularly informative study because IVH occurs closest to the time of collection for the newborn screen. Thus, it was important to show that the metabolic health index was not overly reliant on the presence of IVH for risk stratification performance (fig. S6, A to D). Overall, the metabolic health index was robust to the removal of individual adverse study outcomes from the set of four, suggesting that the model was not overly dependent on the presence of a single adverse outcome for risk stratification.

### Generalization of the metabolic health index in the Ontario cohort

**Fig. 3.**
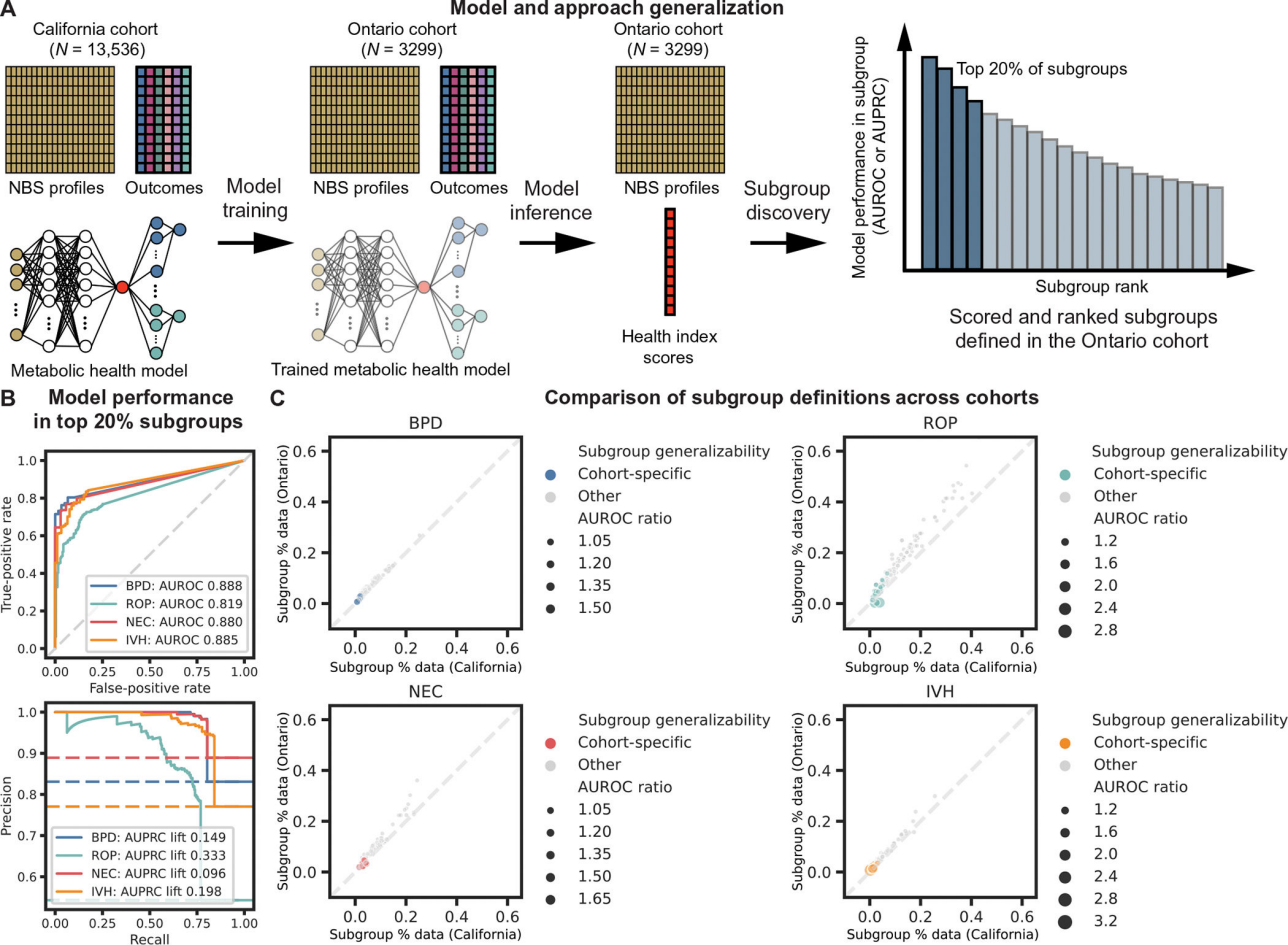
Multicohort generalizability of the metabolic health index model and approach. (**A**) Metabolic health model previously trained using the California cohort was validated on the NSO cohort of 3299 individuals. The resultant health index scores were paired with the subgroup discovery procedure to identify subgroups in the Ontario cohort using metabolite quantiles to define individuals with the strongest model performance. (**B**) ROC curves and precision-recall curves for the metabolic health index model within the top 20% of subgroups. (**C**) Subgroup definitions were compared across the two cohorts in terms of the relative percentage of the dataset encompassed by the subgroup and the within-subgroup AUROC. Scatterplots show each point as one subgroup definition description where point size is relative to the AUROC ratio defined as the subgroup AUROC in California cohort over the subgroup AUROC in the Ontario cohort. Scatterplot axes are the percentage of data captured by each subgroup relative to the total cohort size. Subgroups are colored with cohort-specific labels if the AUROC ratio is within the top 20th percentile.

To investigate the external validity of the metabolic health index in a population drawn from a separate health system, the trained metabolic health index model was applied to the Ontario cohort of 3299 individuals followed by subgroup identification analysis. Subgroup discovery was performed de novo in the Ontario individuals by using metabolite quantiles in the Ontario data to define metabolic health index subgroups (Fig. 3A). Our subgroup discovery procedure was found to be capable of identifying individuals for whom the health index stratified at-risk individuals in the Ontario cohort well with high AUROC and AUPRC (AUROC: BPD: 0.888, ROP: 0.819, NEC: 0.880, IVH 0.885; AUPRC lift over baseline: BPD: 0.149: ROP: 0.333, NEC: 0.096, IVH: 0.198) (Fig. 3B). By matching on subgroup definitions, we were able to evaluate how the same subgroup definition performed across the two cohorts. Although there was some skew in within-subgroup AUROC to the California cohort (fig. S7A), the subgroup definitions were largely concordant in terms of subgroup AUROC, subgroup AUPRC, and size (fig. S7A). The most cohort-specific subgroups (Fig. 3C) and broadly generalizable consensus subgroups (fig. S8A) were identified on the basis of percentile cutoffs of the differences in within-subgroup AUROC between the two cohorts (fig. S7B). Subgroups that had the largest discrepancies in terms of within-subgroup AUROC between California and Ontario tended to have the smallest membership in terms of number of individuals (Fig. 3C). The analysis of the frequency of metabolites used as descriptors in consensus or cohort-specific subgroups did not reveal common high-frequency single metabolites across any outcome (figs. S7C and S8B), suggesting that the metabolic risk model subgroups are not derivatives from one or two broader subgroup definitions (the subgroups represent true metabolic patient heterogeneity). Overall, the subgroup discovery methodology can be applied to additional cohorts for the evaluation and refinement of NBS metabolic health indicators, resulting in generalizable consensus subgroups for model performance.

### Quantification of metabolite-risk model differences between California and Ontario cohorts

**Fig. 4.**
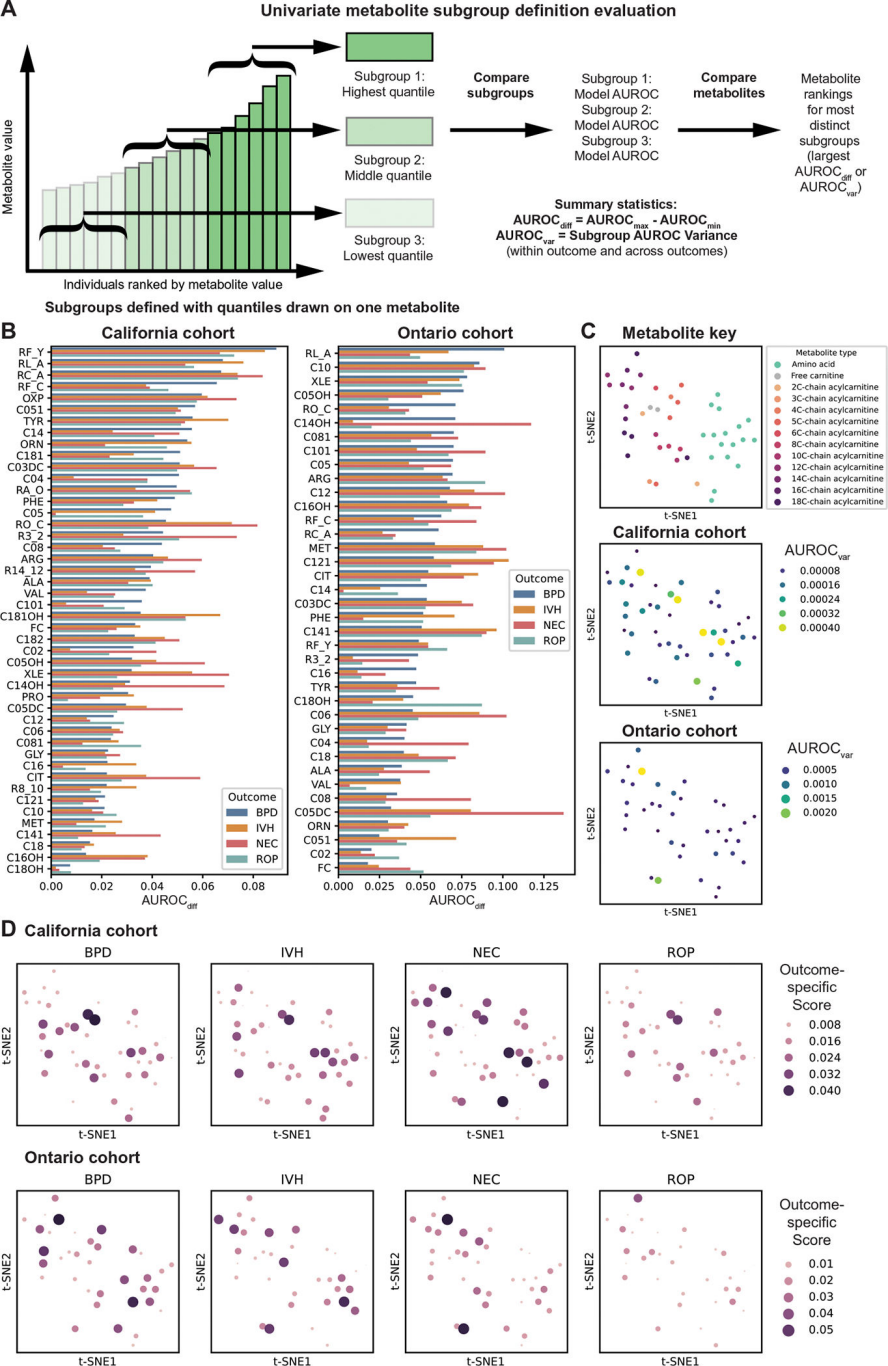
Cohort-specific variability in the relationship between metabolites and metabolic health index subgroups. (**A**) Scoring method for determining univariate metabolite generalizability to compare metabolites across cohorts. Individuals were divided into one of three quantiles for each metabolite value. Individuals in each quantile were scored for model AUROC, and subgroups were quantified with summary statistics such as maximum difference in AUROC between subgroups (AUROC_diff_) or variance in AUROC between subgroups (AUROC_var_). Summary statistics can be calculated across or within the outcomes of prematurity of BPD, ROP, IVH, and NEC. Through summary statistics, metabolites can be ranked for the ability to define metabolic health index subgroups. (**B**) Metabolite rankings in subgroup definition ability through AUROC_diff_ scores. (**C**) Metabolites were embedded in two-dimensional space using t-SNE based on correlations between quantile assignments for individuals in the California cohort. Top: Metabolite key. Middle and bottom panels show AUROCvar scores for the California and Ontario cohorts; each point represents one metabolite colored by AUROC_var_ score. (**D**) Outcome-specific scores defined as the largest difference in AUROC from one outcome versus all other outcomes (a one-versus-all comparison) for subgroups defined using one metabolite.

The previous subgroup discovery results used quantiles in three metabolites to define cross-cohort consensus model performance subgroups. We next asked how individual metabolites may vary in the subgroup discovery definitions across cohorts. To understand potential cohort-specific differences in the importance of metabolites in the risk model, we performed a univariate analysis where individual metabolites were used to define model performance subgroups using three-quantile splits (Fig. 4A). From these univariate metabolite subgroups, we assessed within-subgroup AUROC, within-subgroup AUPRC, and summary statistics across subgroups and across outcomes (Fig. 4A). Individual metabolites were identified that were different across the two cohorts with regard to subgroup definition ability (as quantified by the largest AUROC difference between subgroups of that metabolite) (Fig. 4B). A variance-based summary statistic for the univariate metabolite subgroups also showed distinct cohort differences when compared across all outcomes (Fig. 4C and fig. S9). To identify how the univariate metabolite subgroups varied in each outcome, we derived outcome-specific subgroup scores and compared the metabolites by subgroup definition ability in the California and Ontario cohorts for BPD, IVH, NEC, and ROP separately, finding strong cohort-specific relations between metabolites and health index mode performance (Fig. 4D). These results support the necessity of using multivariate metabolite quantiles specific to individual cohorts or sites to identify consensus subgroups.

## DISCUSSION

We have demonstrated a portable, generalizable metabolic risk stratification approach for adverse outcomes of prematurity built upon a widespread preexisting newborn screen. The metabolic health index developed in this work represents a tool that may be able to aid newborn care management beyond current measures. Risk stratification for BPD and ROP is currently based on physician judgments that take into account GA, exposure to oxygen toxicity, prolonged mechanical ventilation, and presence of systemic infection, alongside their own clinical experience (30, 31). However, these risk assessments are made with implicit subjectivity and biases, the outcome definitions themselves are also subject to misclassification, and diagnosis is largely retrospective. That is, when BPD and ROP are clinically suspected, the disease trajectory has already been well established. Metabolic risk assessment, based on NBS ideally 24 to 48 hours after birth, could potentially inform strategies to increase monitoring, manage clinical exposures, and mitigate prematurity associated sequelae [for example, ventilator pressure/volume strategies in BPD (32, 33), screening ophthalmologic examinations for ROP (34), and ultrasounds and imaging for IVH (35) and NEC (36, 37)]. Stratification of infant risk might eventually lead to risk reduction procedures and protocols that could begin soon after birth, after the turnaround time for routine NBS results (within 48 to 96 hours for sample acquisition, laboratory analysis, and clinical reporting). The proposed deployment for additional adverse outcomes of prematurity must consider the timing of data availability (minutes to hours depending on infrastructure) and model predictions (which are available almost immediately). Therefore, the metabolic health index parallels the time-dependent prevention rationale of NBS programs and has considerable potential to extend the paradigm of NBS in a cost-effective manner.

The challenge of risk stratification for a highly heterogeneous population of very preterm infants required an approach that could handle multiple interacting outcomes and metabolic measurements where the relationships to clinical outcomes were not well established. We addressed this challenge by using a large and unique dataset of very preterm infants with deep clinical profiling and deep learning models to determine complex interactions between metabolic signals to outcomes, as well as the subgroup discovery approach to identify subcohorts with the strongest metabolic biomarker signals of prematurity. We selected deep learning over traditional machine learning methods for several key reasons. First, the bottleneck architecture, which compresses metabolic signals through several layers into a single score, directly addresses a critical clinical reality: In very preterm infants, adverse outcomes rarely occur in isolation. Most infants with one complication also experience others, making risk scores for each outcome less clinically useful than a unified metabolic health index. Second, deep learning’s ability to model complex, nonlinear relationships between metabolites and outcomes is particularly suited to the biological complexity of metabolic biomarker stratification, where interactions between metabolic signals, rather than individual metabolites, are likely to drive clinical risk stratification ability. Third, although traditional ML approaches could address binary prediction tasks, they lack the architectural flexibility to naturally define a shared latent representation (the bottleneck layer) that simultaneously learns from multiple outcomes through multitask learning. Last, deep learning models offer practical advantages for clinical translation: They can be retrained incrementally as new data become available without starting from scratch, can scale efficiently to larger datasets, and can integrate with existing electronic health record deep learning models.

There are several limitations of this work to be addressed in future studies. The metabolic risk models reported herein do not imply a causal link between metabolite profile and clinical disease phenotype. Instead, these metabolite profiles may reflect the underlying biological status or processes that result in vulnerability to adverse outcomes of prematurity. Demographic information was not available for the Ontario cohort, which limited our ability to assess how model performance could be influenced by demographic and race-ethnicity factors, normally a critical part of the development of clinical risk-stratification algorithms. Robust algorithmic solutions to between-group distribution differences are open areas of interest in machine learning and can be applied to the methodological development of metabolic health indicators (38–40). Although the two cohorts in this study represent diverse populations in North America, there may be population risk dynamics that govern adverse neonatal outcomes that vary globally. Despite the broad implementation of NBS programs, linkage between metabolite profiles and neonatal outcomes is not readily available in many clinical settings. Only a few studies have been able to obtain data with NBS metabolites and comprehensive outcome linkage beyond prematurity, GA, and birth-weight (19, 41). Working with local governments and private foundations to generalize the metabolic health index to other populations is of considerable importance and is well aligned with other efforts to expand the health potential of NBS in the global health setting (42). Another aspect of future scaling of the metabolic health index model is the ability to generalize and train models across sites in a way that preserves strict data privacy, which is an active area of research (43–45). Federated learning across sites may therefore be applicable to future multisite development of a general metabolic health index. In this approach, site-specific models are trained and gradients are passed to a central learning model, which then updates the respective weights (46). If applied to metabolite-based risk stratification, then the approach could ensure that no individual-level NBS metabolite information leaves the secure data storage of each site.

The risk of mortality in addition to the other adverse outcomes of prematurity is an important consideration for the development of risk indices in premature newborns (47–49). To address periviable neonates and mortality, a model could be developed that first stratifies infants for overall mortality risk and then determines risk for adverse outcomes of prematurity in the low-mortality subgroup. In this study, the population was limited to preterm infants for predictive power considerations. Future investigations are planned to probe how the relative severity of each adverse outcome and response to clinical interventions for each of the adverse outcomes of prematurity can be factored into metabolic health indicators and to extend metabolic risk stratification to near term and term. Last, the impact of interventions such as total parenteral nutrition, breast milk feeding, transfusion of blood products, or antibiotic administration (to treat sepsis) on model prediction was outside the scope of the current study because the exact timing of NBS sampling was not available (50). Accounting for factors that may influence NBS results is of high interest for the expansion of NBS programs. Longitudinal studies and broader metabolomics in the mother and newborn may elucidate how clinical exposures dynamically affect metabolic profiles (18, 51–56). Discerning the timing of metabolic changes with physiological changes or outcomes may also provide insight into the differential onset of adverse outcomes.

In conclusion, this study demonstrated that very premature newborns can be risk-stratified on the basis of metabolic features obtained from routine NBS screening through data-driven models that use deep learning combined with subgroup discovery refinement. The NBS-based metabolic health index has translational potential to improve treatment decisions in the most vulnerable neonatal patients, given that the required data are readily available as a part of routine neonatal screening in hospitals throughout North America and parts of Europe, Latin America, Asia, and the Pacific.

## MATERIALS AND METHODS

### Study design

Our study objective was to determine whether NBS metabolite profiles contained biomarker signals that could stratify for the most critical adverse conditions of prematurity. To collect these data, we partnered with the CPQCC and the California Department of HCAI (formerly known as the California OSHPD) to align clinical data with newborn analyte screening values stored by the California State Biobank, which in total encompassed 3,175,992 singleton live births in California between 2005 and 2010. We defined our model development cohort by enriching for preterm infants less than 37 weeks of GA from these records with and without major adverse outcomes of prematurity in an age-matched case-control design. Because this was a retrospective study, no blinding was performed with respect to case/control allocation nor with respect to outcome assessment. From the records that included live-born infants who were assessed for the presence of NEC, ROP, and IVH diagnosis codes during hospital stay through clinical record review at time of discharge, analysis was limited to infants between 22 and 29 weeks of GA, which accounted for all infants who had the neonatal outcomes of interest in the study. BPD was defined as infants requiring supplemental oxygen at 36 weeks PMA, where the need for supplemental oxygen was determined by the practices of the individual neonatal intensive care unit, following the cohort definition of BPD case status from a previous study (57). To assess the external validity of the metabolic health index, we identified an independent validation cohort of very preterm and extremely preterm infants where NBS metabolite profiles were linked to clinical outcomes in Ontario, Canada. The study cohorts are described in more detail below.

### Study cohorts

The California State Biobank Cohort consisted of 13,356 preterm singleton live births in California between 2005 and 2010 drawn from the larger cohort of 3,175,992 singleton live births. The cohort was selected for completeness in the clinical record, excluding infants who had missing data for the four diagnoses above and infants for whom there were no metabolic screening data. GA information was categorically encoded in 2-week intervals and BW to 50-g intervals from the source data in accordance with privacy requirements. Neonatal outcomes were defined as infants who had NEC, ROP, BPD, or IVH in either the CPQCC record or the HCAI record for the purpose of analysis, whereas controls for the purposes of model training were defined as individuals without any of the four above outcomes. This study population is unique given a very low population frequency of infants born <29 weeks of gestation along with a very high associated morbidity rate, and thus there is a relatively small number of controls—further enrichment of controls in this study would not realistically be achievable because these individuals were sampled across the entire state of California (birth population of ~500,000 births/year) to obtain the current number of controls within this GA range. The number of individuals for the least frequent outcome of NEC (1439 cases to 12,097 controls) supported a power of 0.948 to detect an effect of Cohen’s *d* = 0.1 at a significance level of α = 0.05 using a two-tailed t test. For individuals born from the years of 2005 to November of 2009, all newborns had bloodspot collections for NBS performed between 12 hours and 8 days after birth. Data on timing of collection were not available for infants born after December 2009 in the dataset. Metabolite values were available for 47 metabolites in the NBS dataset after dropping metabolites that had missing values in more than 30% of infants. These NBS metabolites and neonatal outcomes formed the primary variables used in our analyses. Data regarding blood transfusions and missed newborn screens were unavailable. The study was approved by the Stanford University Institutional Review Board (IRB) [protocol #24543, principal investigator (PI): G.M.S.] and by the California Health and Human Services Agency Committee for the Protection of Human Subjects (project #12–11–0906, PI: G.M.S.).

The NSO cohort was composed of 3299 preterm infants less than or equal to a GA of 32 weeks drawn from a larger NSO cohort that contains the NBS metabolite profiles corresponding to more than 1 million individual infants in Ontario between January 2010 and December 2014. Infants who screened positive for any disorders on the NBS panel or who were transfused before blood spot collection were excluded from the original cohort. Individual NBS profiles were linked to infant sex, BW, GA, some limited demographic information, and clinical outcomes through the DAD populated and maintained by the Canadian Institute of Health Information (CIHI). The presence of BPD, IVH, NEC, or ROP was determined by matching on ICD codes in the preterm infant cohort. ICD codes used to match individuals for each adverse outcome were BPD: P27.1; IVH: P52.1, P52.21, P52.22; NEC: P77, P77.1, P77.2, P77.3; ROP: H35.1. For the least frequent outcome of NEC (303 cases and 2996 controls), the sample size of this cohort has power of 0.913 to detect an effect of Cohen’s *d* = 0.2 at a significance level of α = 0.05 for a two-tailed t test. For comparison with California NBS profiles, 38 metabolites were matched as 100% complete in all cohort individuals between the two cohorts for analysis. Because of data privacy restrictions enforcing the minimal presence of individually identifiable information in the source dataset, race and ethnicity data were not available for this iteration of the Ontario cohort. Data regarding timing of NBS sample collection, blood transfusions, and missed newborn screens were not available for this cohort. Data access and management were conducted in collaboration with the University of Ottawa and the Ottawa Hospital Research Institute (OHRI). The study identification number assigned to this collaborative project between Stanford University and OHRI was TRIM 0901 323 000.

### Metabolic health index model development

The multitask deep neural network architectures in this study took metabolite values as input before two subsequent hidden layers [100 hidden units each with rectified linear unit (ReLU) activation] before individual modules (one per outcome of interest) that consisted of one hidden layer (100 hidden units with ReLU activation) and a single output. Deep learning models were implemented in PyTorch (58) and trained for 50 epochs using Adam optimization [learning_rate = 0.001, betas = (0.9,0.999), eps = 1 × 10^−08^, weight_decay = 0], early stopping (where training was stopped after five epochs without improvement), and binary cross-entropy loss. Deep neural network models were trained using repeated 10-fold cross-validation with further evaluation using a holdout validation set not used during the training process that consisted of 25% of the total dataset. Through-out the manuscript, the training set and test set refer to split datasets throughout repeated K-fold cross-validation. The term holdout validation set refers to the subset of the data that was not used during repeated K-fold training, which was selected using a train/test split strategy without stratification. For evaluating model predictions, the mean model predictions corresponding to the epoch with the lowest test loss across all iterations was used. Repeated K-fold splitting was performed using scikit-learn (59). Model training was performed with PyTorch or PyTorch Lightning (60) with models checkpointed on lowest validation loss. Bottleneck neural network architectures were designed with a bottleneck layer [linear (input dimensions to bottleneck dimensions) → ReLU → linear (bottleneck dimensions to next layer dimensions] after the two hidden layers immediately following the input metabolite values. We varied the number of hidden units in the bottleneck layer from 1 to 20 units. The bottleneck layer then fed into the four adverse outcome-specific modules as described above. The bottleneck layer outputs were also evaluated directly as a means to compress NBS metabolite information into a single metabolic health index in a one hidden unit bottleneck layer. The output from the single bottleneck unit model is referred to as the metabolic health index throughout the text. Outcome ablation experiments were performed by iteratively removing one outcome from the set of outcomes (BPD, IVH, NEC, and ROP) while leaving three outcomes present for the multitask model with a single bottleneck layer to be used as the metabolic health index.

### Subgroup discovery

Subgroup discovery was used to determine biological patient subpopulations in which the algorithm has a higher performance. Given a dataset X and search space S, which provides candidate descriptions of relevant subgroups, subgroup discovery uses the target of interest T and a set of selection criteria C to find and rank subgroups (27, 61). Subgroup discovery was performed using an extension of the pysubgroup package (62) with the following components: (i) NBS data X, where discretized values of individual NBS metabolites composed the search space S (discretized into 2, 3, and 5 quantiles); (ii) ground truth Y and metabolic health index model output $\hat{Y}$ were used to construct the AUROC as the target of interest T (where infants without any of the adverse outcomes of prematurity were compared against infants with the various adverse outcomes of prematurity); and (iii) a modification of the standard subgroup quality function which integrates the AUROC of a subgroup as the selection criterion C (63). Because neural network activations do not inherently have a direction relative to the outcome label, the health index was occasionally constructed by reversing the bottleneck layer activations a using $1-\frac{a-a_{\text{min}}}{a_{\text{max}}-a_{\min}}$, so that the resultant AUROC corresponds with identifying individuals who were at lower risk of adverse outcomes. The standard quality function defined a family of interestingness measures that trades off the relative size (i.e., generality) of subgroups versus the difference between the target shares in the subgroup and the overall dataset through the parameter α (26, 63). In practice, the selection of a balanced subgroup size with subgroup enrichment of target T. The values of a used for subgroup discovery for each adverse outcome using the metabolic health index were BPD: 0.0575, ROP: 0.073, IVH: 0.0585, NEC: 0.085. Values for α were empirically chosen to balance subgroup size versus target enrichment and avoid trivial subgroups. Each individual analysis was found to be affected differently by α, thus necessitating specific values per analysis of each adverse outcome. The subgroup analysis relied on the combination of descriptors in the search space to generate candidate subgroups. Because of the computational complexity of an exhaustive search strategy, beam search was used as a heuristic search method, and the number of descriptors for each candidate subgroup was set to four (64). For the outcome ablation experiments, subgroup discovery was performed where the subgroup discovery procedure still had access to outcome labels for all four adverse outcomes of prematurity. This was done because the subgroup discovery procedure requires knowledge of which individuals have none of the four adverse outcomes of prematurity for accurate scoring of AUROC without the presence of comorbidities. We performed de novo subgroup discovery in the Ontario cohort using quantile definitions defined based on metabolite values for the Ontario cohort only. The independent subgroup discovery results were then merged based on subgroup definitions (i.e., description of the subgroup based on quantiles, which is provided in a standardized way), which allowed for a cross-cohort comparison of subgroups. We compared model performance for subgroups across sites using the AUROC performance ratio

$$\text{Subgroup}\;\text{performance}\;\text{ratio}\equiv \frac{{\text{AUROC}}_{\text{California}}}{{\text{AUROC}}_{\text{Ontario}}}$$

Thus, a ratio of 1 would be a subgroup in which the within-subgroup health index AUROC performance is the same in both cohorts and a larger AUROC ratio shows subgroups that are more cohort specific for the California cohort. Cohort-specific subgroups were defined as those subgroups that had an absolute residual AUROC defined as $\left|{\text{AUROC}}_{\text{California}}-{\text{AUROC}}_{\text{Ontario}}\right|$ greater than the 90th percentile or larger than 0.2 as an empirical threshold. Consensus subgroups were labeled with the criterion of an AUROC performance ratio below the 20th percentile of absolute values (corresponding to the closest AUROC values between California and Ontario).

### Single metabolite generalizability analysis

Reducing the subgroup discovery problem to a subproblem where subgroups are defined using quantiles from one metabolite was performed to explore the generalizability of the metabolite-model performance relationship from one cohort to another. Individual metabolite values were split into three-quantile splits within each cohort to define three subgroups per metabolite. Each of these univariate metabolite subgroups was then scored on the basis of within-subgroup model AUROC and within-subgroup AUPRC. The three univariate metabolite subgroups were also quantified using summary statistics, defined as ${\text{AUROC}}_{\text{diff}}=\left|{\text{AUROC}}_{\text{max}}-{\text{AUROC}}_{\text{min}}\right|$ among the three univariate subgroups and ${\text{AUROC}}_{\text{Var}}$ as the variance in AUROC for the three subgroups. These summary statistics quantify how distinct the subgroups are defined for each metabolite (a score of 0 would correspond to complete equivalence of all subgroups). Each of these summary statistics was reported per outcome (BPD, IVH, NEC, and ROC) and reported agnostic of outcome for visualization purposes. Last, we defined an outcome-specific score as the absolute difference in the maximum AUROC value for the three univariate subgroups in one outcome subtracted from the minimum AUROC value for the three univariate subgroups in all other outcomes. This metric is designed to quantify how well the metabolites distinguish subgroups of one outcome versus all other outcomes in a one-versus-rest comparison. For a given outcome i and AUROC vector A representing univariate subgroup AUROC values matching certain outcomes, this outcome-specific score is defined as

$${\text{score}}_{i}={\text{max}}_{\text{outcome}=i}\;{\text{A}}_{i}-{\text{min}}_{\text{outcome}=j,\;j\neq i}\;{\text{A}}_{j}$$

### Statistical analysis

Comparisons of demographics and clinical variables were performed using the scipy (65) implementation of Fisher’s exact test for categorical variables (with the Monte Carlo method for contingency tables greater than 2 × 2), where P values are reported for selected clinical variables. Other statistical comparisons between continuous variables were performed using two-sided t tests or Mann-Whitney U tests depending on whether the data met test assumptions for normality. Significance levels were reported for several values of α, with the first significance threshold considered at α = 0.05 (after any corrections for multiple comparisons). Correlations between continuous variables were quantified using Spearman’s ρ. Significantly correlated metabolites in the correlation networks were determined by a Bonferroni-corrected threshold for the two-sided Fisher’s asymptotic P value. Individuals within the top percentile target of subgroups were compared to those outside of the top percentile to determine a statistically significant difference in BW distributions within each GA range category using the scipy implementation of the nonparametric Mann-Whitney U test with Bonferroni-corrected P values. Because the data only contained GA ranges and BW ranges, BW values were taken to be in the middle of the given BW range for the numerical statistical tests. Confidence intervals were calculated as the means ± 2 SDs, corresponding to the normal-based 95% confidence interval.

## Supplementary Material

Tables and Figures

Supplementary Materials

The PDF file includes:

Materials and Methods

Figs. S1 to S9

Tables S1 to S10

References (67–80)

Other Supplementary Material for this manuscript includes the following:

MDAR Reproducibility Checklist

## Figures and Tables

**Table 1.** Description of the California State Biobank cohort (*N* = 13,356).

Category	Controls	Cases
*Neonatal condition*		
BPD	9347	4189*
IVH	9276	4260*
NEC	12,097	1439*
ROP	7818	5718*
Any of the above	1440	11,916*
*Maternal race/ethnicity*		
Hispanic	2309^†^	4896^‡^
Non-Hispanic white	993^†^	1805^‡^
Non-Hispanic Black	579^†^	1237^‡^
Asian	416^†^	911^‡^
Pacific Islander	25^†^	72^‡^
American Indian/Alaskan Native	19^†^	47^‡^
Missing	66^†^	147^‡^
*Newborn sex*		
Male	2362^†^	4885^‡^
Female	2048^†^	4241^‡^

* Case numbers include infants with multiple conditions.

† Control numbers refer to newborns without BPD, IVH, NEC, or ROP.

‡ Case numbers refer to newborns with BPD, IVH, NEC, or ROP.

**Table 2.** Description of the Newborn Screening Ontario cohort (*N* = 3299). This cohort includes 1811 male infants [635 controls (19.25%); 1176 cases (35.65%)] and 1488 female infants [547 controls (16.6%); 941 cases (28.52%)]. SD, standard deviation; CI, confidence interval.

Neonatal condition	Controls (N, newborns without BPD, IVH, NEC, or ROP)	Mean GA of controls (weeks ± SD)	Cases (N, includes infants with multiple conditions)	Mean GA of cases (weeks ± SD)	Difference in means (95% CI)
BPD	2683	28.07 ± 1.84	616	26.64 ± 1.77	1.43 (1.20, 1.66)
IVH	2484	28.02 ± 1.82	815	27.09 ± 2.02	0.94 (0.70, 1.17)
NEC	2996	27.88 ± 1.89	303	27.19 ± 1.98	0.69 (0.35, 1.02)
ROP	1568	28.24 ± 1.88	1133	27.29 ± 1.80	0.94 (0.76, 1.12)
Any of the above	1182	28.59 ± 1.68	2117	27.73 ± 1.88	1.26 (1.08, 1.43)

## Data Availability

Preexisting data access policies outlined by the California State Biobank, the California Department of HCAI, and the CPQCC govern data access requests. Requests will be reviewed by the steering committees from each organization before providing access. NBS data in Ontario are under the purview of the ICES and are governed by preexisting data use and access policies. The dataset from this study is held securely in coded form at ICES. Although legal data-sharing agreements between ICES and data providers (health care organizations and government) prohibit ICES from making the dataset publicly available, access may be granted to those who meet prespecified criteria for confidential access, available at www.ices.on.ca/DAS (email: das@ices.on.ca). The full dataset creation plan and underlying analytic code are available from the authors upon request, understanding that the computer programs may rely upon coding templates or macros that are unique to ICES and are therefore either inaccessible or may require modification. Model training code, model prediction results, data analysis results, and checkpointed versions of models for independent analysis of new datasets are available at Zenodo (https://doi.org/10.5281/zenodo.17984155) (66).
