# Supplementary material for: Quantitative assessment of neonatal health using dried blood spot metabolite profiles and deep learning
Source: Sci Transl Med. Author manuscript; Available in PMC 2026 Jun 9. (PMC13246279; doi:10.1126/scitranslmed.adv4942)
Supplement: Tables and Figures [file NIHMS2155580-supplement-Tables_and_Figures.pdf]

Science Translational Medicine
AAAS

Supplementary Materials for

Quantitative assessment of neonatal health using dried blood spot metabolite profiles and deep learning

Alan L. Chang *et al.*

Corresponding author: Gary M. Shaw, gmshaw@stanford.edu; Karl G. Sylvester, karls@stanford.edu; Nima Aghaeepour, naghaeep@stanford.edu

Sci. Transl. Med. **18**, eadv4942 (2026)
DOI: 10.1126/scitranslmed.adv4942

The PDF file includes:

Materials and Methods
Figs. S1 to S9
Tables S1 to S10
References (*67–80*)

Other Supplementary Material for this manuscript includes the following:

MDAR Reproducibility Checklist

Supplementary Materials and Methods

Construction of correlation networks

A correlation graph of metabolites was constructed by first calculating the Spearman correlation between all measured metabolite values in the NBS data. Each metabolite was a node in the correlation graph and edges were drawn between significantly correlated metabolites (by two-sided Fisher's asymptotic P value < 0.05 calculated using the corPvalueFisher function from the `WGCNA` package) (*67*). The absolute value correlation matrix R was then projected into two-dimensional space using t-stochastic nearest neighbors embedding (t-SNE) (*68*) where we used $1 - R$ as the distance matrix as input so that two perfectly correlated metabolites would have a distance of 0. For changes in correlation coefficients between cases and controls, the adjusted correlation difference was calculated as $\rho_{\text{cases}} + \rho_{\text{controls}}$ when Spearman correlation coefficients were of opposite signs and $\rho_{\text{cases}} - \rho_{\text{controls}}$ otherwise. Absolute correlation difference was calculated as $|\rho_{\text{cases}} - \rho_{\text{controls}}|$. Statistical significance of correlation disruption between cases and controls was assessed using Fisher's Z transformation test, with correction for multiple comparisons using the Benjamini-Hochberg false discovery rate (FDR) method at $\alpha = 0.05$.

Model evaluation

Models were evaluated using the area under the precision-recall curve (AUPRC) in order to account for class imbalance. Precision is defined as *Precision ≡ TP / (TP + FP)* where TP denotes true positives and FP denotes false positives. Recall is defined as *Recall ≡ TP / (TP + FN)* where TP denotes true positives and FN denotes false negatives. AUPRC was calculated using the trapezoidal rule under the operating points defined as a pair (R_n, P_n), which are the precision and recall values at the nth threshold. In the scoring scheme, only infants who were negative for all four neonatal outcomes were considered as negative cases. AUPRC was calculated using pr_auc from the `yardstick` package (*69*) in R. Precision-recall curves were drawn using the `yardstick` and `ggplot2` (*70*) packages in R. For model evaluation within

python, the `scikit-learn` function average_precision_score was used to calculate AUPRC. Average precision is calculated as $\Sigma_n(R_n - R_{n-1})P_n$ where P_n and R_n are the precision and recall at the nth threshold and is an alternative to the trapezoidal rule for AUPRC (*71*). AUPRC scores were normalized relative to frequency as (AUPRC / Frequency) for comparison across outcomes, referred to in the text as "AUPRC Lift" or "AUPRC Gain".

Comparison between the metabolic health index and other machine learning models

Random forest (*72*), histogram-based gradient boosting classifier (*73*), linear regression, elastic net (*74*), and logistic regression models were trained using the `scikit-learn` framework. Hyperparameter tuning for random forest and histogram gradient boosting classifier models was performed using randomized search within a 3-fold inner cross validation loop for 100 iterations. The hyperparameter grid for random forest models included a number of estimators of 50, 100, 200, 400, 600, 800, 1000, 1400, 1800, 2000, maximum depth of 10, 30, 50, 70, 90, 100, or None, and minimum sample split of 2, 5, and 10. For the histogram gradient boosting classifier the hyperparameter grid included a learning rate of 0.001, 0.1, 0.5, 1.0, L2 regularization of 0.0, 0.1, 1.0, 5.0, 10 and 50, and minimum samples per leaf of 10, 20, 50, and 100. XGBoost (*75*) models were trained using the scikit-learn-compatible API through the `xgboost` Python package. The randomized search hyperparameter grid for XGBoost included the number of estimators as above for random forest, learning rates of 0.001, 0.01, 0.1, 0.2, 0.3, subsample fractions of 0.5, 0.6, 0.7, 0.8, 0.9, and 1.0, minimum child weight of 0.1, 1, 3, 5, 7, and 10, maximum depth of 6, 10, 15, and 20, gamma of 0.0, 0.25, 0.5, 1.0, and regression lambda values of 0.1, 1, 5, 10, 50, and 100. Hyperparameter tuning for elastic net logistic regression models was done with nested K-Fold cross validation and grid-search over the L1 ratio range of 0.1, 0.5, 0.7, 0.8, 0.9, 0.95, 0.99, and 1.0. For Lasso models, hyperparameter tuning was performed using grid search over 20 C values.

Feature importance

Univariate calculation of metabolite importance for outcomes was evaluated based on AUPRC, where the metabolite values were used directly to calculate AUPRC for each outcome of interest. Metabolites with high AUPRC reflect the case where higher values of the metabolite are associated with the neonatal outcome of interest. Statistical significance of AUPRC values was assessed using permutation testing (n = 1,000 permutations) with FDR correction for multiple comparisons. Significance testing for enrichment of univariate AUPRC with analyte categories was performed using Fisher's exact test as implemented in `scipy`. The mean AUPRC across all four neonatal outcomes was then used as the overall feature importance ranking. Neural network models were then trained iteratively using this feature importance ranking, removing the lowest ranked feature remaining at each iteration. Calculation of feature importance for deep learning models was performed using the captum package (*76*), using feature permutation (*77*), integrated gradients (*78*), and DeepLiftSHAP (*79*, *80*).

SUPPLEMENTARY FIGURES

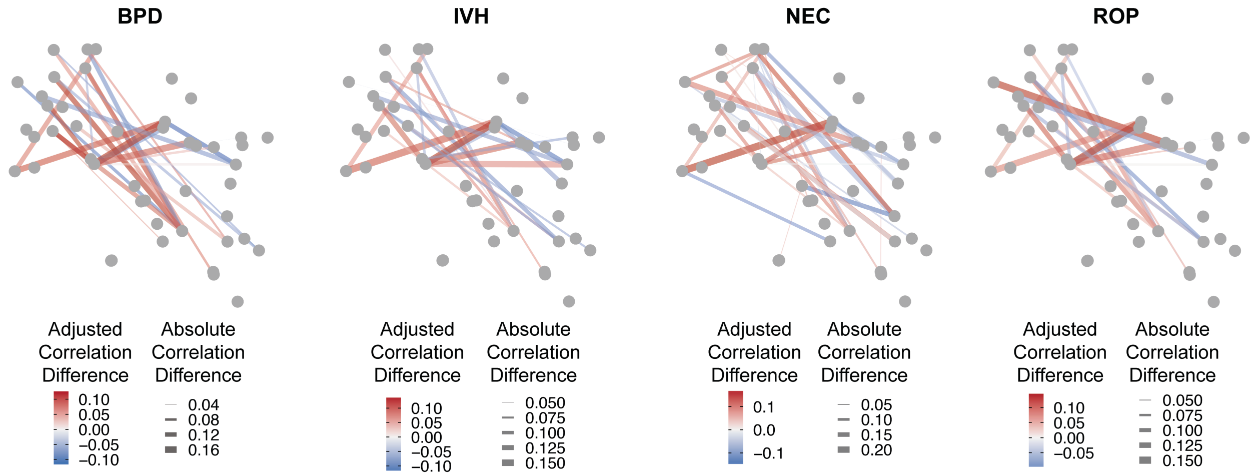

Figure S1. Adverse outcomes of prematurity are associated with specific changes in NBS metabolite pairs. Spearman correlation between all NBS metabolites was calculated in infants with BPD, IVH, NEC, ROP, and in infants without. The results are visualized in correlation networks where nodes represent metabolites embedded in two-dimensional space using t-SNE and edges are drawn only for those metabolite pairs that change directionality of the Spearman correlation coefficient when comparing pairwise correlation in infants with neonatal conditions compared to infants without any of the above outcomes. Edge thickness corresponds to the absolute value of the difference in correlation coefficients. Adjusted correlation difference refers to Spearman correlation in infants with neonatal conditions adjusted using the correlation coefficient in control infants.

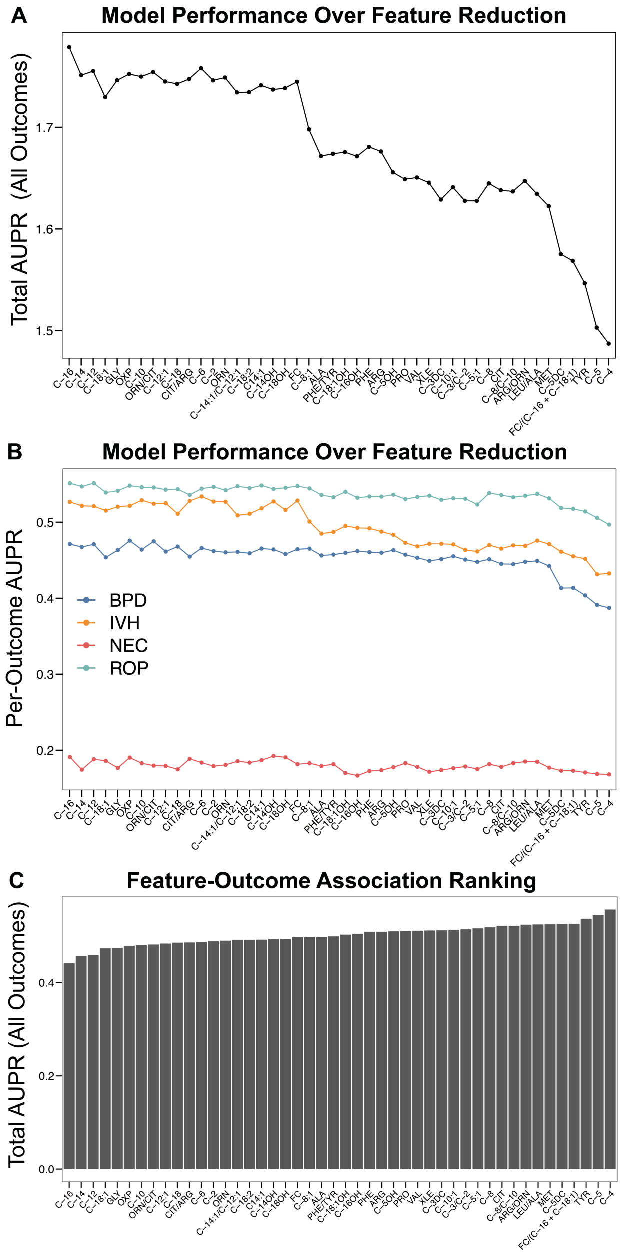

Figure S2. Identification of the strongest NBS metabolite contributions to overall model performance. Metabolite features were ranked in terms of total AUPRC across each neonatal outcome of interest and a deep multitask model (without a bottleneck layer) was trained through an iterative feature removal procedure where the lowest ranking feature in terms of AUPRC was removed at each iteration. This procedure was repeated until only one feature remained. (A) Model performance for all neonatal outcomes depicted as total AUPRC summed across all neonatal outcomes. Points represent the mean over five iterations of repeated K-Fold cross validation. (B) Model performance for each neonatal outcome in terms of AUPRC. (C) AUPRC feature ranks from lowest individual metabolite AUPRC to highest.

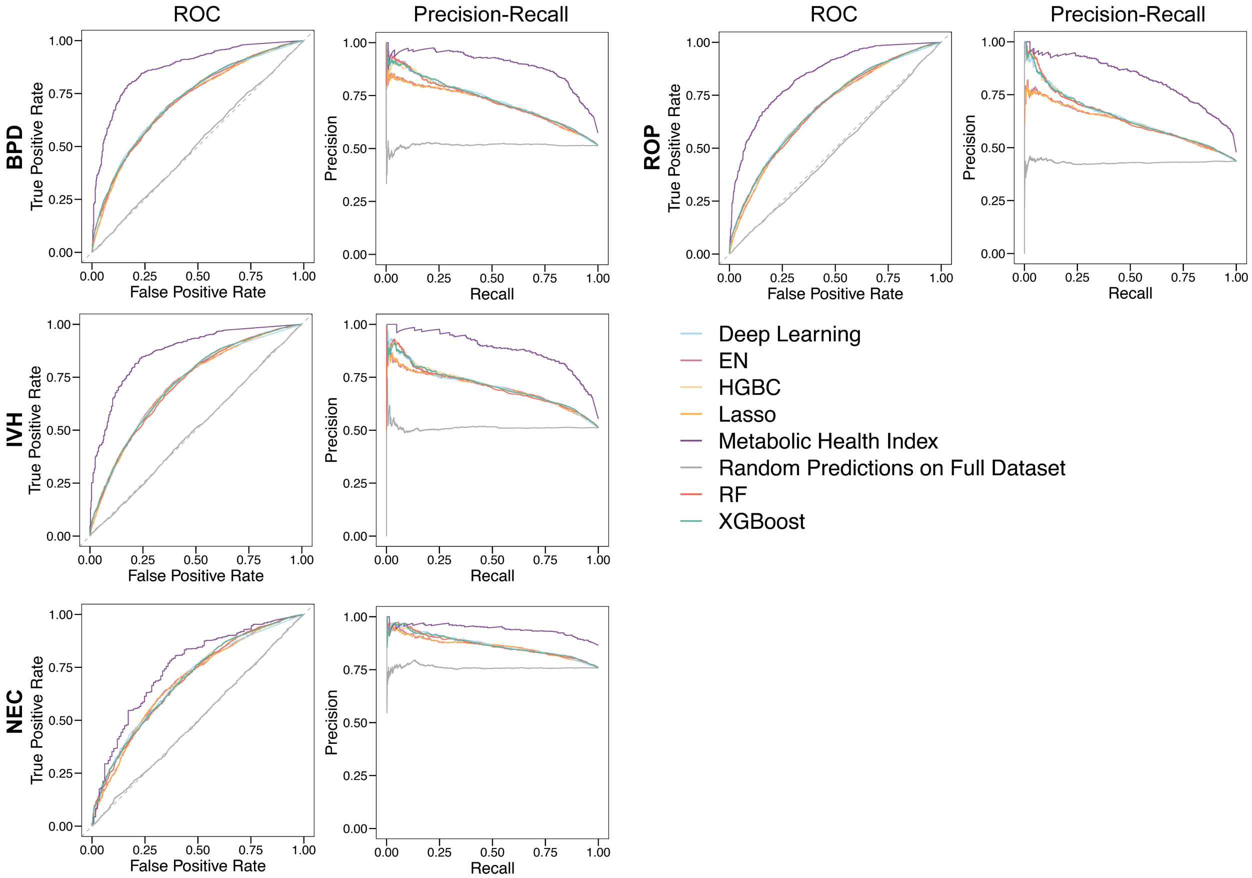

Figure S3. The metabolic health index outperforms other machine learning approaches. Elastic net (EN), Lasso, random forest (RF), deep learning, Histogram Gradient Boosting Classifier (HGBC), and XGBoost models were trained to differentiate healthy infants from those with any of the four adverse outcomes of BPD, IVH, NEC, or ROP. ROC and precision-recall curves are shown for the ability of single risk indicators to separate healthy infants from infants with the conditions of BPD, IVH, NEC, and ROP. As a baseline, random uniform predictions on the full dataset were used to draw ROC and precision-recall curves.

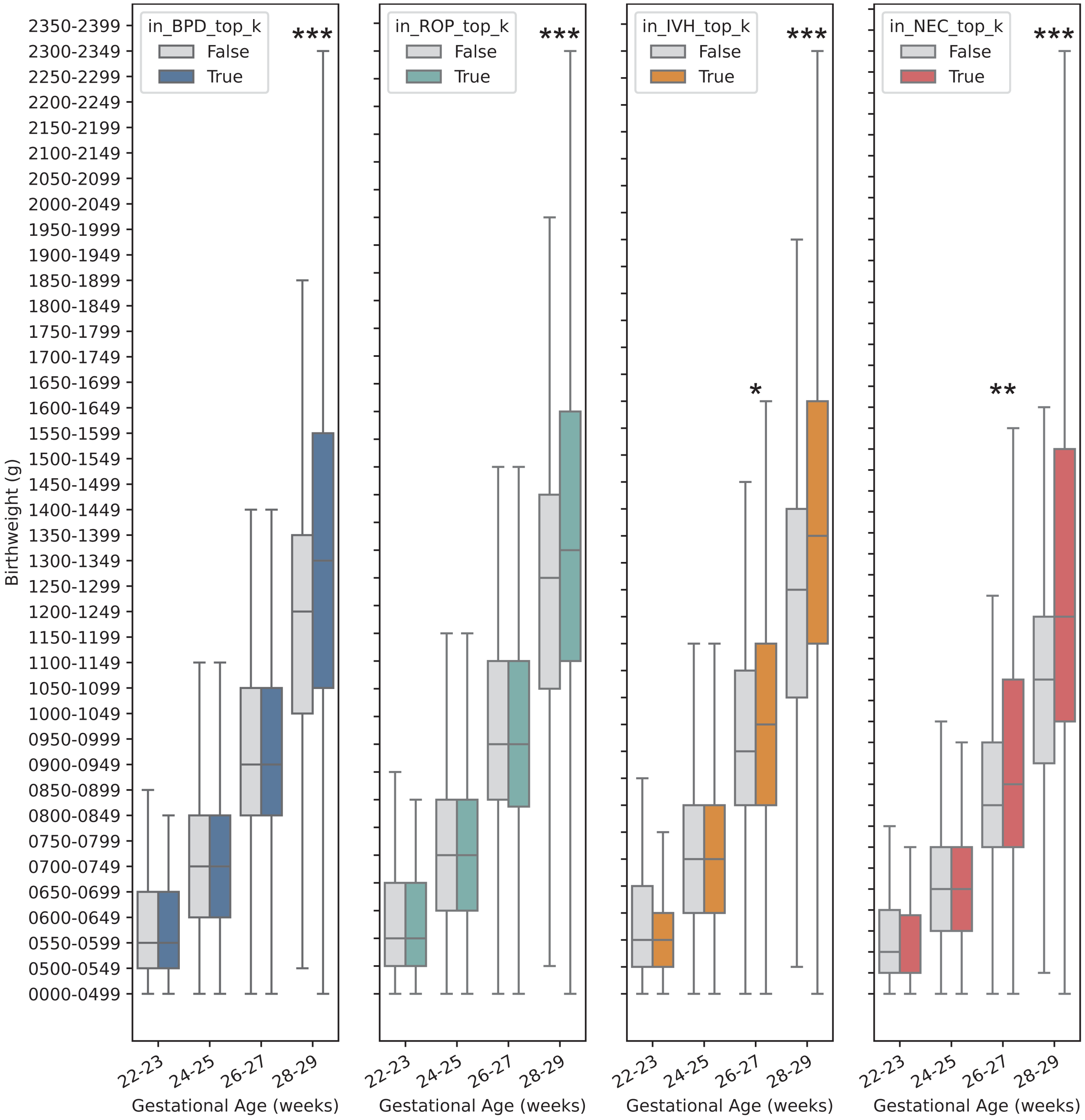

Figure S4. Top subgroups for metabolic health index performance do not trivially capture top percentiles of birthweight or gestational age. Boxplot analysis of gestational age ranges and birthweight ranges for subgroups identified by the subgroup discovery process in the four study outcomes of BPD, ROP, IVH, and NEC. The gray bars represent individuals who are not in the top 20th target percentile of subgroups for model performance whereas colored bars represent individuals who are within the top 20th target percentile. *P < 0.05, **P < 0.01, ***P < 0.001 by Mann-Whitney *U* Test.

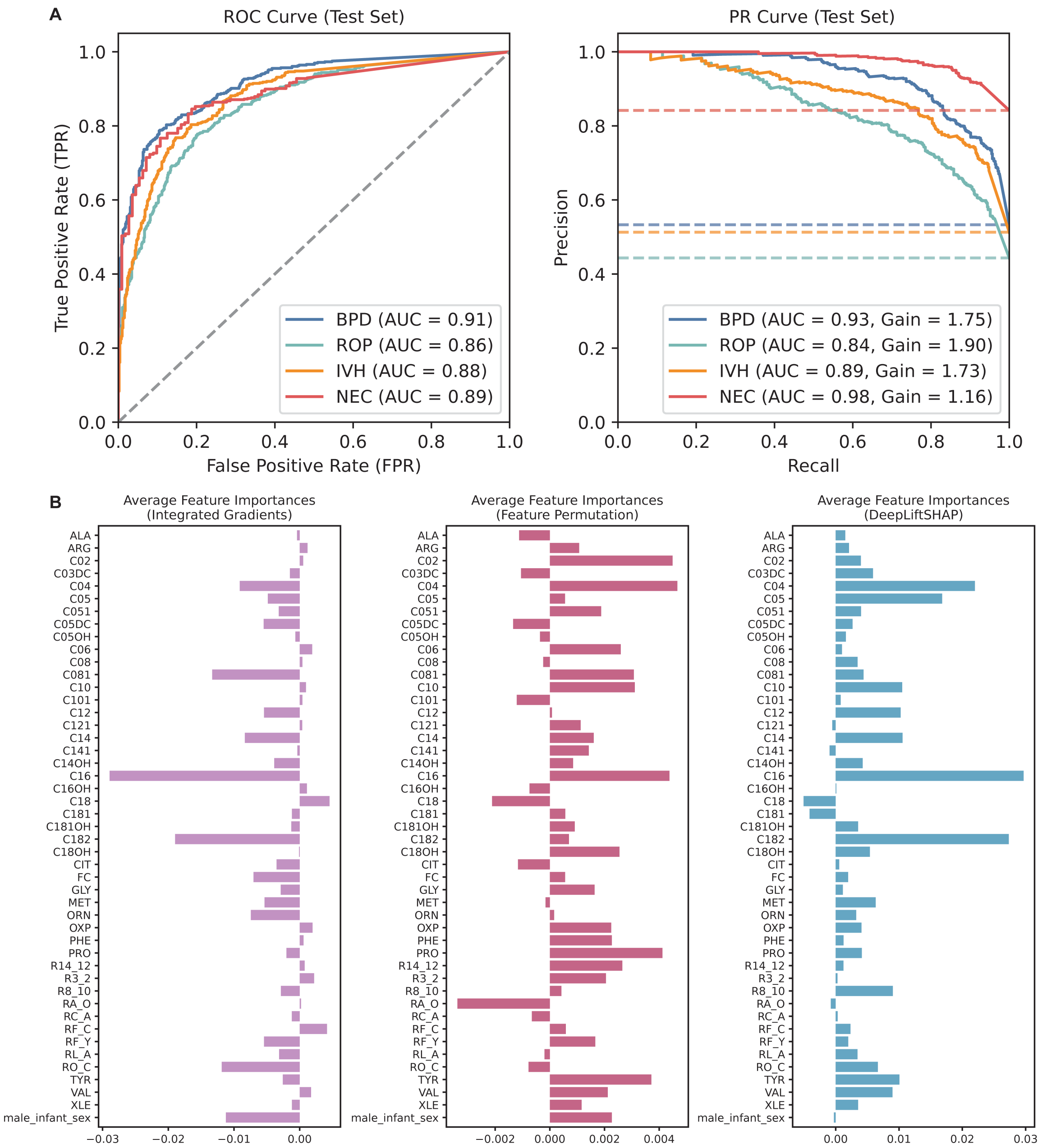

Figure S5. Integration of infant sex as a clinical risk variable into the metabolic health index. Male infant sex was encoded as a clinical risk variable included alongside the newborn screen metabolites as an input to a metabolic health index model as before. (A) ROC and precision-recall curves are shown for the ability of the metabolic health index to separate healthy infants from infants with the conditions of BPD, IVH, NEC, and ROP within the top 20% of subgroups. (B) Feature importance analysis by integrated gradients, feature permutation, and DeepLiftSHAP for the newborn screen metabolites and infant sex.

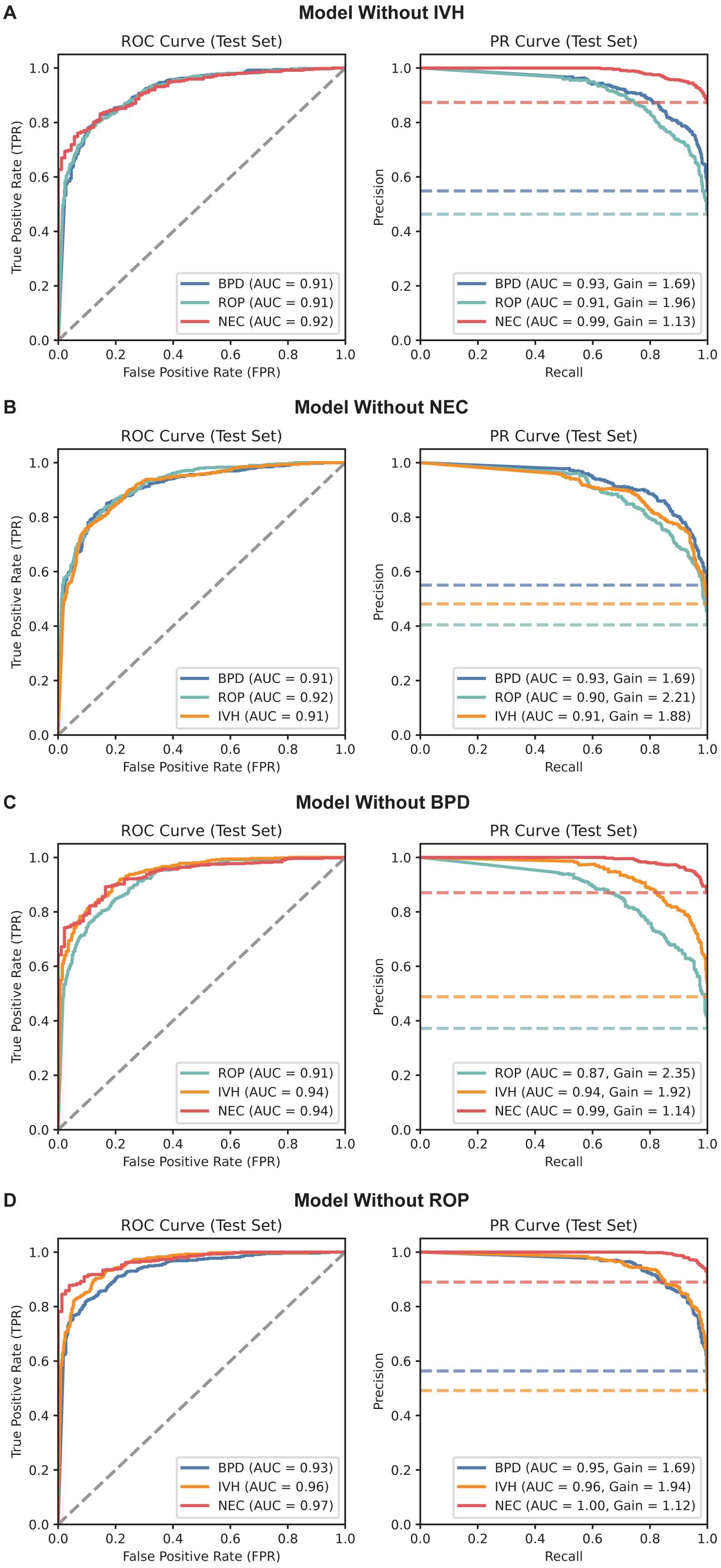
A
Model Without IVH
ROC Curve (Test Set)
True Positive Rate (TPR)
False Positive Rate (FPR)
BPD (AUC = 0.91)
ROP (AUC = 0.91)
NEC (AUC = 0.92)
PR Curve (Test Set)
Precision
Recall
BPD (AUC = 0.93, Gain = 1.69)
ROP (AUC = 0.91, Gain = 1.96)
NEC (AUC = 0.99, Gain = 1.13)
B
Model Without NEC
ROC Curve (Test Set)
True Positive Rate (TPR)
False Positive Rate (FPR)
BPD (AUC = 0.91)
ROP (AUC = 0.92)
IVH (AUC = 0.91)
PR Curve (Test Set)
Precision
Recall
BPD (AUC = 0.93, Gain = 1.69)
ROP (AUC = 0.90, Gain = 2.21)
IVH (AUC = 0.91, Gain = 1.88)
C
Model Without BPD
ROC Curve (Test Set)
True Positive Rate (TPR)
False Positive Rate (FPR)
ROP (AUC = 0.91)
IVH (AUC = 0.94)
NEC (AUC = 0.94)
PR Curve (Test Set)
Precision
Recall
ROP (AUC = 0.87, Gain = 2.35)
IVH (AUC = 0.94, Gain = 1.92)
NEC (AUC = 0.99, Gain = 1.14)
D
Model Without ROP
ROC Curve (Test Set)
True Positive Rate (TPR)
False Positive Rate (FPR)
BPD (AUC = 0.93)
IVH (AUC = 0.96)
NEC (AUC = 0.97)
PR Curve (Test Set)
Precision
Recall
BPD (AUC = 0.95, Gain = 1.69)
IVH (AUC = 0.96, Gain = 1.94)
NEC (AUC = 1.00, Gain = 1.12)

Figure S6. Relative contribution of each adverse outcome of prematurity to metabolic health index model performance. Ablation experiments were performed by iteratively removing one of the adverse outcomes of prematurity during model training, leaving three outcomes for metabolic health index model training. Following the training procedure, subgroup discovery was performed as before with access to all four adverse outcome labels to properly label control infants. ROC curves and precision-recall (PR) curves are shown for the resultant model performance in the top 20th target percentile of subgroups for health index models (A) without IVH, (B) without NEC, (C) without BPD, and (D) without ROP.

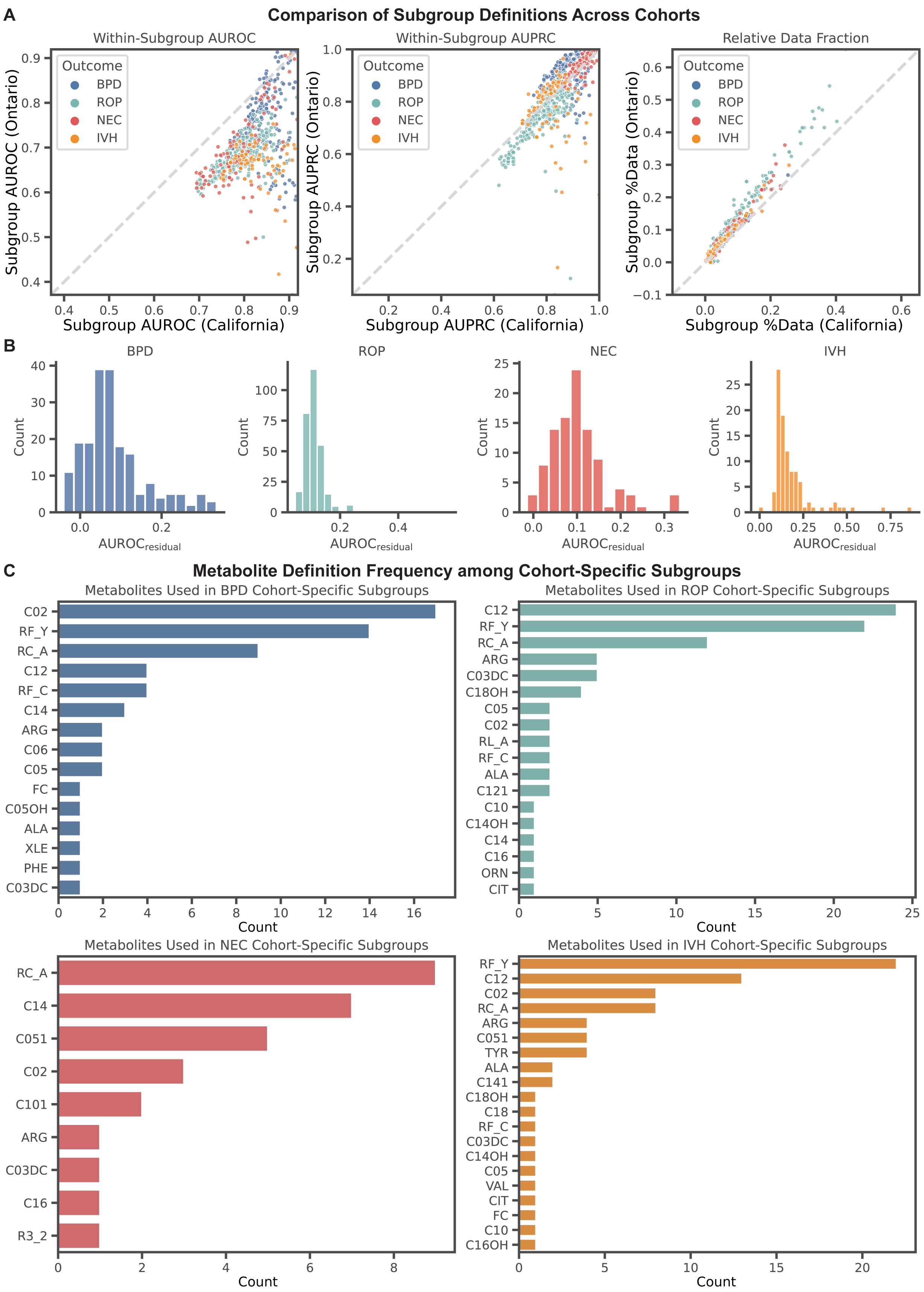
A
Comparison of Subgroup Definitions Across Cohorts
Within-Subgroup AUROC
Subgroup AUROC (Ontario)
Subgroup AUROC (California)
Within-Subgroup AUPRC
Subgroup AUPRC (Ontario)
Subgroup AUPRC (California)
Relative Data Fraction
Subgroup %Data (Ontario)
Subgroup %Data (California)
Outcome
BPD
ROP
NEC
IVH
B
BPD
ROP
NEC
IVH
Count
AUROC$_{residual}$
C
Metabolite Definition Frequency among Cohort-Specific Subgroups
Metabolites Used in BPD Cohort-Specific Subgroups
C02
RF_Y
RC_A
C12
RF_C
C14
ARG
C06
C05
FC
C05OH
ALA
XLE
PHE
C03DC
Count
Metabolites Used in ROP Cohort-Specific Subgroups
C12
RF_Y
RC_A
ARG
C03DC
C18OH
C05
C02
RL_A
RF_C
ALA
C121
C10
C14OH
C14
C16
ORN
CIT
Count
Metabolites Used in NEC Cohort-Specific Subgroups
RC_A
C14
C051
C02
C101
ARG
C03DC
C16
R3_2
Count
Metabolites Used in IVH Cohort-Specific Subgroups
RF_Y
C12
C02
RC_A
ARG
C051
TYR
ALA
C141
C18OH
C18
RF_C
C03DC
C14OH
C05
VAL
CIT
FC
C10
C16OH
Count

Figure S7 (related to Fig. 3). Additional subgroup comparison metrics and metabolite frequencies for cohort-specific subgroups. (A) Comparisons of within subgroup-AUROC, within-subgroup AUPRC, and relative percentage of data for subgroup definitions matched across the California and Ontario cohorts. Each point represents one subgroup description in one outcome, dashed line represents where the metric would be identical in both cohorts. (B) Histograms showing the distribution of the $AUROC_{residual}$ scores that are used to label cohort-specific subgroups in Fig. 3C. Cohort-specific subgroups are labeled based on $AUROC_{residual}$ percentiles at the edge of the distributions, i.e., subgroups whose AUROC vary the most between the California and Ontario cohorts for the same subgroup definition. (C) Frequency of metabolites used in the definitions for cohort-specific subgroups in each adverse outcome of prematurity.

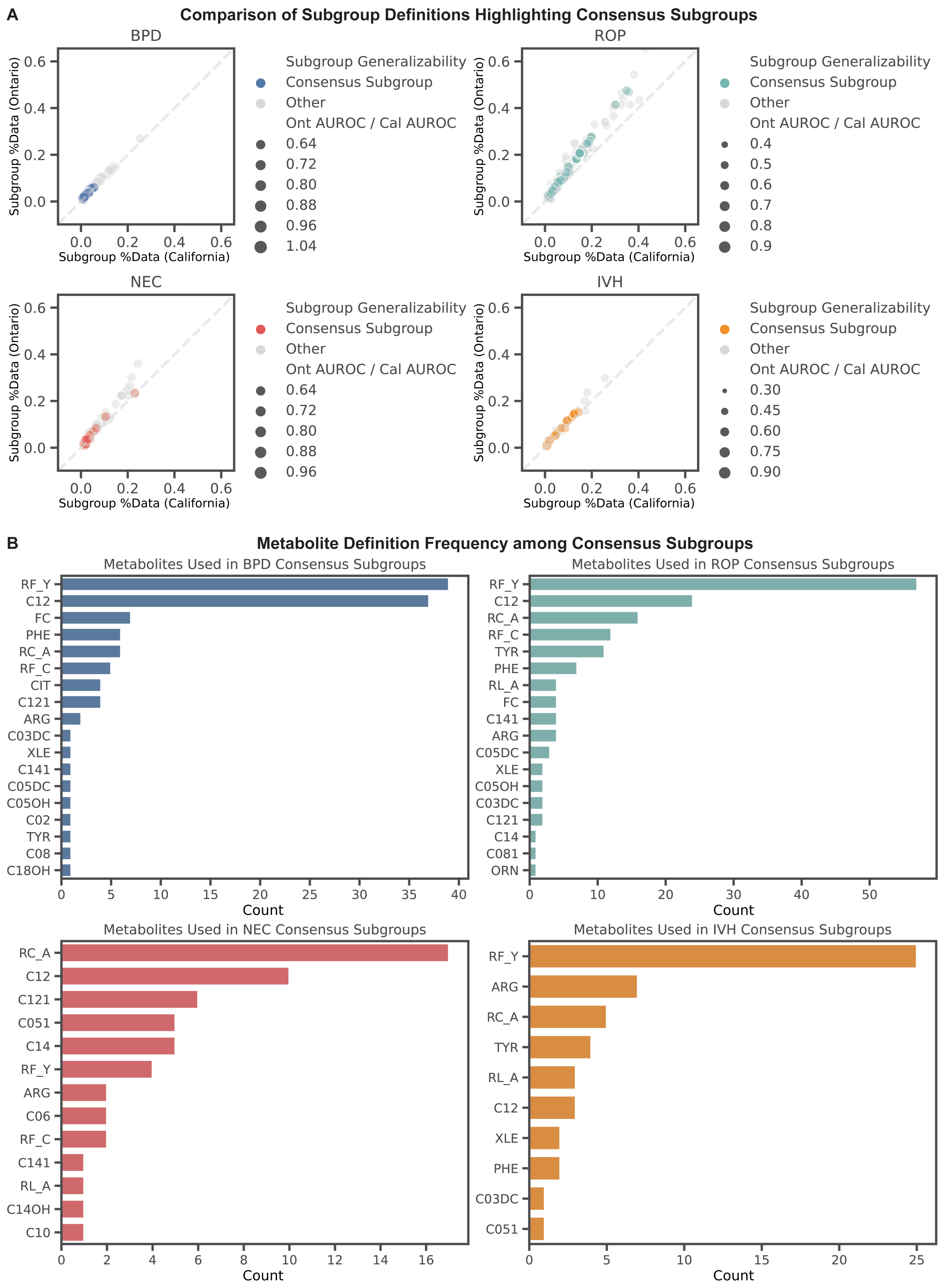
A
Comparison of Subgroup Definitions Highlighting Consensus Subgroups
BPD
ROP
NEC
IVH
Subgroup %Data (Ontario)
Subgroup %Data (California)
Subgroup Generalizability
Consensus Subgroup
Other
Ont AUROC / Cal AUROC
0.64
0.72
0.80
0.88
0.96
1.04
0.4
0.5
0.6
0.7
0.8
0.9
0.30
0.45
0.60
0.75
0.90
B
Metabolite Definition Frequency among Consensus Subgroups
Metabolites Used in BPD Consensus Subgroups
RF_Y
C12
FC
PHE
RC_A
RF_C
CIT
C121
ARG
C03DC
XLE
C141
C05DC
C05OH
C02
TYR
C08
C18OH
Metabolites Used in ROP Consensus Subgroups
RF_Y
C12
RC_A
RF_C
TYR
PHE
RL_A
FC
C141
ARG
C05DC
XLE
C05OH
C03DC
C121
C14
C081
ORN
Metabolites Used in NEC Consensus Subgroups
RC_A
C12
C121
C051
C14
RF_Y
ARG
C06
RF_C
C141
RL_A
C14OH
C10
Metabolites Used in IVH Consensus Subgroups
RF_Y
ARG
RC_A
TYR
RL_A
C12
XLE
PHE
C03DC
C051
Count

Figure S8 (related to Fig. 3). Identification of consensus subgroups across California and Ontario cohorts. (A) Consensus subgroups identified between the California and Ontario cohorts based on AUROC ratio quantifying relative within-subgroup performance for the same subgroup quantile definitions applied to the two cohorts independently. Here the size of the point is scaled to the ratio of the within-subgroup AUROC in Ontario over the within-subgroup AUROC in California so that the values closest to 1.0 will correspond to consensus subgroups. Axes show the subgroup size relative to the entire cohort, expressed as a percentage. Dashed line represents where the relative subgroup data percentage would be identical in both cohorts. Panels are separated by each adverse outcome of prematurity. (B) Frequency of metabolites used in the definitions for consensus subgroups in each adverse outcome of prematurity.

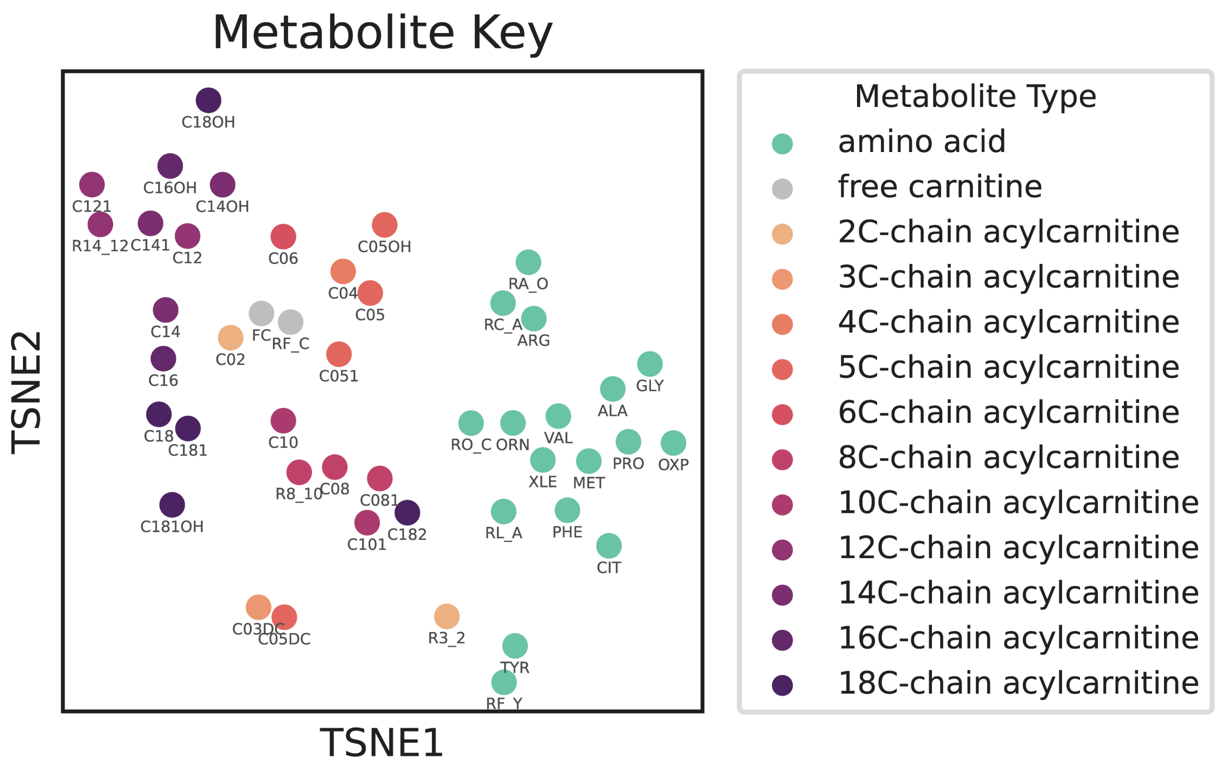

Figure S9 (related to Fig. 4C). Labeled metabolite layout key for univariate subgroup experiments. Metabolites were embedded in two-dimensional space using t-SNE based on correlations between quantile assignments for individuals in the California cohort. Each point represents a metabolite that is common to both California and Ontario NBS metabolite cohorts. Individual text labels for each metabolite are shown for completeness.

SUPPLEMENTARY TABLES

Table S1. Univariate metabolite feature area under the precision-recall curve (AUPRC) for each neonatal outcome of interest.

Metabolite	BPD AUPRC	IVH AUPRC	NEC AUPRC	ROP AUPRC	Total AUPRC
C-4	0.565	0.576	0.307	0.636	2.084
C-5	0.553	0.548	0.307	0.630	2.038
Tyrosine (TYR)	0.531	0.552	0.284	0.606	1.974
FC / (C-16 + C-18:1)	0.532	0.529	0.278	0.601	1.940
C-5DC	0.527	0.531	0.276	0.598	1.933
Citrulline (CIT)	0.527	0.537	0.263	0.600	1.926
Leucine / Alanine Ratio (LEU/ALA)	0.535	0.503	0.271	0.600	1.909
C-8/C-10	0.520	0.515	0.263	0.600	1.898
C-8	0.509	0.512	0.270	0.593	1.884
Methionine (MET)	0.492	0.531	0.261	0.583	1.867
C-5:1	0.513	0.509	0.257	0.588	1.867
C-3DC	0.507	0.503	0.262	0.585	1.856
C-16OH	0.503	0.509	0.260	0.578	1.850
C-5OH	0.494	0.493	0.266	0.585	1.838
C-3/C-2	0.493	0.510	0.250	0.579	1.832
C-10:1	0.491	0.491	0.266	0.580	1.828
Arginine / Ornithine Ratio (ARG/ORN)	0.488	0.519	0.255	0.566	1.828
Valine (VAL)	0.492	0.506	0.249	0.574	1.822
Citrulline / Arginine Ratio (CIT/ARG)	0.502	0.497	0.240	0.573	1.813
C-18:1OH	0.492	0.497	0.244	0.572	1.805
C14:1	0.493	0.495	0.245	0.562	1.795
C-14OH	0.490	0.497	0.245	0.559	1.792
Leucine or Isoleucine (XLE)	0.482	0.496	0.244	0.568	1.790
C-14:1/C-12:1	0.493	0.488	0.237	0.563	1.782

C-18OH	0.481	0.487	0.250	0.562	1.780
Proline (PRO)	0.469	0.509	0.238	0.558	1.775
C-6	0.480	0.489	0.238	0.560	1.767
Phenylalanine (PHE)	0.472	0.498	0.237	0.558	1.765
Free Carnitine (FC)	0.482	0.467	0.241	0.559	1.749
Arginine (ARG)	0.465	0.486	0.235	0.552	1.738
C-12:1	0.464	0.479	0.237	0.545	1.725
C-8:1	0.468	0.456	0.236	0.557	1.716
5-oxoproline (OXP)	0.461	0.473	0.223	0.547	1.704
C-10	0.455	0.467	0.232	0.538	1.693
Phenylalanine \ Tyrosine Ratio (PHE/TYR)	0.462	0.455	0.222	0.539	1.679
C-2	0.461	0.442	0.227	0.544	1.674
Alanine (ALA)	0.443	0.479	0.218	0.528	1.668
Ornithine (ORN)	0.448	0.450	0.218	0.540	1.656
C-18	0.456	0.439	0.209	0.542	1.646
Glycine (GLY)	0.444	0.455	0.214	0.532	1.644
C-12	0.443	0.447	0.221	0.520	1.631
C-18:2	0.439	0.431	0.220	0.532	1.621
C-18:1	0.435	0.430	0.206	0.523	1.594
C-14	0.436	0.433	0.208	0.513	1.590
Ornithine / Citrulline Ratio (ORN/CIT)	0.425	0.426	0.212	0.519	1.584
C-16	0.402	0.398	0.187	0.485	1.471

Table S2. Metabolite pair correlation coefficient sign changes within each neonatal outcome.

Metabolite Pair		Spearman Rho (Infants with Outcome)	Spearman Rho P Value (Infants with Outcome)	Spearman Rho -log10 P Value (Infants with Outcome)	Spearman Rho (Infants without Outcome)	Spearman Rho P Value (Infants without Outcome)	Spearman Rho -log10 P Value (Infants without Outcome)	Absolute Difference in Spearman Rho	Neonatal Outcome
ARG/ORN	OXP	0.101	0.000	10.211	-0.024	0.110	0.960	0.125	BPD
C-10	ARG	0.114	0.000	12.933	-0.008	0.599	0.223	0.122	BPD
C-12:1	ARG	0.077	0.000	6.165	-0.005	0.755	0.122	0.081	BPD
C-16	C-5	0.005	0.728	0.138	-0.116	0.000	14.057	0.122	BPD
C-8/C-10	PHE	-0.003	0.854	0.068	0.053	0.000	3.397	0.056	BPD
CIT/ARG	C-10	-0.101	0.000	10.345	0.016	0.290	0.538	0.117	BPD
CIT/ARG	C14:1	-0.006	0.716	0.145	0.069	0.000	5.372	0.075	BPD
CIT	C14:1	0.073	0.000	5.592	-0.027	0.074	1.130	0.099	BPD
LEU/ALA	C-10	0.001	0.966	0.015	-0.095	0.000	9.598	0.096	BPD
ORN	C-10	0.127	0.000	15.806	-0.010	0.522	0.283	0.137	BPD
ORN	C-14OH	0.013	0.406	0.392	-0.076	0.000	6.344	0.089	BPD
ORN/CIT	C-10	0.086	0.000	7.534	-0.024	0.118	0.928	0.109	BPD
ORN/CIT	C-14OH	0.001	0.963	0.016	-0.067	0.000	5.083	0.068	BPD
OXP	C-5	0.125	0.000	15.438	-0.026	0.084	1.078	0.151	BPD
PHE	C-10	0.102	0.000	10.492	-0.020	0.178	0.749	0.122	BPD
PHE/TYR	ARG	-0.066	0.000	4.752	0.011	0.474	0.324	0.077	BPD
PHE/TYR	C-16	0.045	0.003	2.463	-0.037	0.015	1.827	0.082	BPD
PHE/TYR	C-2	-0.024	0.119	0.923	0.057	0.000	3.796	0.081	BPD
PHE/TYR	C-4	-0.111	0.000	12.283	0.035	0.021	1.674	0.146	BPD
PHE/TYR	C-5	-0.026	0.087	1.060	0.148	0.000	22.499	0.175	BPD
PHE/TYR	FC/(C-16 + C-18:1)	-0.035	0.024	1.623	0.128	0.000	16.772	0.162	BPD
PHE/TYR	PHE	-0.003	0.855	0.068	0.121	0.000	15.248	0.124	BPD
TYR	C-18:1	-0.032	0.036	1.446	0.005	0.726	0.139	0.038	BPD
XLE	C-16	0.024	0.126	0.898	-0.098	0.000	10.198	0.122	BPD
XLE	PHE/TYR	-0.003	0.869	0.061	0.113	0.000	13.334	0.116	BPD
ARG/ORN	OXP	0.091	0.000	8.621	-0.024	0.110	0.960	0.115	IVH
C-10	ARG	0.097	0.000	9.638	-0.008	0.599	0.223	0.105	IVH

C-12:1	ARG	0.070	0.000	5.371	-0.005	0.755	0.122	0.075	IVH
C-14	C-5	0.018	0.236	0.628	-0.100	0.000	10.481	0.118	IVH
C-16	C-5	0.001	0.951	0.022	-0.116	0.000	14.057	0.117	IVH
CIT/ARG	C-10	-0.078	0.000	6.446	0.016	0.290	0.538	0.094	IVH
CIT	C14:1	0.065	0.000	4.714	-0.027	0.074	1.130	0.092	IVH
FC/(C-16 + C-18:1)	ORN	-0.004	0.790	0.102	0.073	0.000	5.952	0.077	IVH
LEU/ALA	C-18	0.005	0.740	0.131	-0.041	0.007	2.178	0.046	IVH
ORN	C-10	0.081	0.000	6.914	-0.010	0.522	0.283	0.091	IVH
ORN	C14:1	0.009	0.554	0.256	-0.100	0.000	10.567	0.109	IVH
ORN/CIT	C-10	0.043	0.005	2.301	-0.024	0.118	0.928	0.067	IVH
OXP	C-5	0.136	0.000	18.469	-0.026	0.084	1.078	0.162	IVH
PHE	C-10	0.060	0.000	4.061	-0.020	0.178	0.749	0.080	IVH
PHE/TYR	ARG	-0.074	0.000	5.827	0.011	0.474	0.324	0.084	IVH
PHE/TYR	C-16	0.111	0.000	12.360	-0.037	0.015	1.827	0.147	IVH
PHE/TYR	C-4	-0.101	0.000	10.474	0.035	0.021	1.674	0.136	IVH
PHE/TYR	C-5	-0.012	0.452	0.344	0.148	0.000	22.499	0.160	IVH
PHE/TYR	FC/(C-16 + C-18:1)	-0.027	0.078	1.106	0.128	0.000	16.772	0.155	IVH
TYR	C-18:1	-0.079	0.000	6.565	0.005	0.726	0.139	0.084	IVH
VAL	C14:1	0.001	0.960	0.018	-0.084	0.000	7.562	0.084	IVH
VAL	C-14OH	0.004	0.819	0.087	-0.074	0.000	6.057	0.077	IVH
XLE	C-16	0.017	0.280	0.552	-0.098	0.000	10.198	0.115	IVH
XLE	PHE/TYR	-0.018	0.249	0.603	0.113	0.000	13.334	0.131	IVH
ARG/ORN	C-14	0.008	0.765	0.116	-0.122	0.000	15.489	0.130	NEC
ARG/ORN	C-8:1	-0.003	0.921	0.036	0.061	0.000	4.265	0.063	NEC
ARG/ORN	ORN	-0.010	0.700	0.155	0.076	0.000	6.370	0.086	NEC
ARG/ORN	OXP	0.097	0.000	3.620	-0.024	0.110	0.960	0.121	NEC
C-10	ARG	0.113	0.000	4.757	-0.008	0.599	0.223	0.121	NEC
C-12:1	ARG	0.075	0.004	2.349	-0.005	0.755	0.122	0.080	NEC
C-12	ARG	0.051	0.055	1.259	-0.134	0.000	18.380	0.184	NEC
C-14:1/C-12:1	C-2	-0.011	0.680	0.168	0.044	0.003	2.487	0.055	NEC
C-14	C-5	0.073	0.005	2.268	-0.100	0.000	10.481	0.173	NEC

C-16	C-5	0.005	0.837	0.077	-0.116	0.000	14.057	0.122	NEC
C-18:2	C-12	0.006	0.824	0.084	-0.147	0.000	21.963	0.152	NEC
C-18:2	C-5DC	-0.002	0.953	0.021	0.048	0.002	2.815	0.049	NEC
C-8/C-10	OXP	0.003	0.900	0.046	-0.116	0.000	13.943	0.119	NEC
CIT/ARG	C-10	-0.083	0.002	2.780	0.016	0.290	0.538	0.099	NEC
CIT/ARG	C-12	-0.002	0.949	0.023	0.145	0.000	21.614	0.147	NEC
CIT/ARG	C-3/C-2	0.007	0.804	0.095	-0.054	0.000	3.526	0.061	NEC
CIT	C14:1	0.132	0.000	6.338	-0.027	0.074	1.130	0.159	NEC
LEU/ALA	ARG/ORN	-0.007	0.799	0.097	0.114	0.000	13.434	0.120	NEC
LEU/ALA	C-16OH	0.008	0.758	0.120	-0.066	0.000	4.925	0.074	NEC
LEU/ALA	FC/(C-16 + C-18:1)	-0.035	0.191	0.719	0.142	0.000	20.651	0.177	NEC
ORN	C-10	0.112	0.000	4.717	-0.010	0.522	0.283	0.122	NEC
ORN/CIT	C-10	0.043	0.105	0.978	-0.024	0.118	0.928	0.066	NEC
ORN/CIT	FC	0.000	0.999	0.000	0.044	0.004	2.427	0.044	NEC
OXP	C-5	0.199	0.000	13.653	-0.026	0.084	1.078	0.225	NEC
PHE	C-10	0.064	0.015	1.832	-0.020	0.178	0.749	0.085	NEC
PHE/TYR	ARG	-0.038	0.154	0.812	0.011	0.474	0.324	0.048	NEC
PHE/TYR	C-16	0.037	0.165	0.782	-0.037	0.015	1.827	0.073	NEC
PHE/TYR	C-4	-0.079	0.003	2.572	0.035	0.021	1.674	0.114	NEC
PHE/TYR	C-5	-0.018	0.499	0.302	0.148	0.000	22.499	0.166	NEC
PHE/TYR	FC/(C-16 + C-18:1)	-0.007	0.790	0.102	0.128	0.000	16.772	0.135	NEC
TYR	C-18:1	-0.028	0.287	0.542	0.005	0.726	0.139	0.033	NEC
VAL	C14:1	0.062	0.020	1.708	-0.084	0.000	7.562	0.145	NEC
XLE	C-16	0.028	0.291	0.536	-0.098	0.000	10.198	0.126	NEC
XLE	PHE/TYR	-0.010	0.698	0.156	0.113	0.000	13.334	0.123	NEC
ARG/ORN	OXP	0.078	0.000	8.478	-0.024	0.110	0.960	0.102	ROP
C-10	ARG	0.087	0.000	10.324	-0.008	0.599	0.223	0.095	ROP
C-12:1	ARG	0.062	0.000	5.623	-0.005	0.755	0.122	0.067	ROP
CIT/ARG	C-10	-0.066	0.000	6.274	0.016	0.290	0.538	0.082	ROP
CIT	C14:1	0.061	0.000	5.400	-0.027	0.074	1.130	0.088	ROP
LEU/ALA	FC/(C-16 + C-18:1)	-0.003	0.845	0.073	0.142	0.000	20.651	0.145	ROP

ORN	C-10	0.105	0.000	14.756	-0.010	0.522	0.283	0.115	ROP
ORN	C14:1	0.001	0.964	0.016	-0.100	0.000	10.567	0.101	ROP
ORN/CIT	C-10	0.069	0.000	6.823	-0.024	0.118	0.928	0.093	ROP
OXP	C-5	0.113	0.000	17.138	-0.026	0.084	1.078	0.139	ROP
PHE	C-10	0.066	0.000	6.292	-0.020	0.178	0.749	0.087	ROP
PHE/TYR	ARG	-0.076	0.000	8.098	0.011	0.474	0.324	0.087	ROP
PHE/TYR	C-16	0.042	0.002	2.793	-0.037	0.015	1.827	0.078	ROP
PHE/TYR	C-2	-0.008	0.536	0.271	0.057	0.000	3.796	0.065	ROP
PHE/TYR	C-4	-0.111	0.000	16.563	0.035	0.021	1.674	0.146	ROP
PHE/TYR	C-5	-0.003	0.812	0.090	0.148	0.000	22.499	0.152	ROP
PHE/TYR	FC/(C-16 + C-18:1)	-0.017	0.189	0.724	0.128	0.000	16.772	0.145	ROP
TYR	C-18:1	-0.036	0.007	2.185	0.005	0.726	0.139	0.041	ROP
VAL	C14:1	0.010	0.440	0.357	-0.084	0.000	7.562	0.094	ROP
XLE	C-16	0.012	0.364	0.439	-0.098	0.000	10.198	0.110	ROP
XLE	PHE/TYR	-0.002	0.859	0.066	0.113	0.000	13.334	0.115	ROP

Table S3: Performance of multitask machine learning classifiers on neonatal outcomes.

Evaluation Metric	Neonatal Outcome	Deep Multitask Architecture (K-Fold CV Combined Test Set) (± standard deviation)	Deep Multitask Architecture (Holdout Validation Set) (± standard deviation)	Deep Multitask Architecture (Top Ranked Subgroups Comprising 20% of K-Fold CV Test Set) (± standard deviation)	Deep Multitask Architecture (Top Ranked Subgroups Comprising 20% of Holdout Validation Set) (± standard deviation)
AUPRC	BPD	0.672 (±.0088)	0.681 (±.0034)	0.810 (±.0152)	0.713 (±.0063)
	IVH	0.688 (±.0085)	0.678 (±.0034)	0.823 (±.0170)	0.697 (±.0039)
	NEC	0.373 (±.0099)	0.403 (±.0043)	0.576 (±.0432)	0.442 (±.0118)
	ROP	0.710 (±.0069)	0.727 (±.0021)	0.829 (±.0141)	0.763 (±.0010)

Deep multitask models were trained using a repeated K-fold cross validation procedure and validated on a hold-out validation set. The mean and standard deviation of AUPRC values are shown over 10 iterations of repeated K-fold cross-validation. Subgroup discovery was performed to identify infant subpopulations with increased predictive performance. Subgroups were identified using the K-fold CV test set. The same subgroups were then applied to the holdout validation set and ranked. The mean and standard deviation of AUPRC values are shown for model performance over 10 iterations of repeated K-fold cross validation in top-ranked subgroups that represent 20% of all extremely preterm infants in either the K-fold cross validation test set or the holdout validation set.

Table S4: Performance single-unit bottleneck metabolic health index for discerning healthy newborns from those with neonatal outcomes. (related to Fig. 2)

Evaluation Metric	Neonatal Outcome	1-unit Bottleneck Output as Metabolic Health Index (K-Fold CV Combined Test Set) (± standard deviation)	1-unit Bottleneck Output as Metabolic Health Index (Holdout Validation Set) (± standard deviation)	1-unit Bottleneck Output as Metabolic Health Index (Top Ranked Subgroups Comprising 20% of K-Fold CV Test Set) (± standard deviation)	1-unit Bottleneck Output as Metabolic Health Index (Top Ranked Subgroups Comprising 20% of Holdout Validation Set) (± standard deviation)
AUPRC	BPD	0.700 (±.0044)	0.727 (±.0027)	0.896 (±.0056)	0.773 (±.0054)
	IVH	0.685 (±.0045)	0.685 (±.0028)	0.883 (±.0070)	0.745 (±.0016)
	NEC	0.860 (±.0037)	0.850 (±.0028)	0.979 (±.0026)	0.903 (±.0031)
	ROP	0.614 (±.0045)	0.621 (±.0028)	0.805 (±.0080)	0.721 (±.0035)

Deep multitask bottleneck models were trained using a repeated K-fold cross validation procedure and validated on a hold-out validation set using a single bottleneck unit as a metabolic health index which was evaluated on the ability to distinguish infants without the four adverse outcomes of prematurity. Mean and standard deviation of AUPRC values on the combined test set folds and on the holdout validation set are shown over 10 iterations of repeated K-fold cross-validation. Subgroup discovery was performed to identify infant subpopulations further improved stratification of infants without adverse outcomes. The subgroups were identified on the K-fold cross validation test set. Mean and standard deviation of AUPRC values are shown for model performance over 10 iterations of repeated K-fold cross validation in top-ranked subgroups that represent 20% of all extremely preterm infants in either the K-fold cross validation test set or the holdout validation set.

Table S5. Correlation between NBS metabolites and the metabolic health index.

Metabolite	Abbreviated Label	Description	Category	Spearman Rho (California Cohort)	Spearman Rho (Ontario Cohort)
ALA	ALA	Alanine	amino acid	0.178	0.283
ARG	ARG	Arginine	amino acid	0.082	0.165
C-2	C02	C-2	short-chain acylcarnitine	0.194	0.204
C-3DC	C03DC	C-3DC	short-chain acylcarnitine	-0.097	0.007
C-4	C04	C-4	short-chain acylcarnitine	-0.368	-0.197
C-5	C05	C-5	short-chain acylcarnitine	-0.262	-0.189
C-5:1	C051	C-5:1	short-chain acylcarnitine	-0.060	-0.064
C-5DC	C05DC	C-5DC	short-chain acylcarnitine	-0.202	-0.125
C-5OH	C05OH	C-5OH	short-chain acylcarnitine	-0.006	-0.021
C-6	C06	C-6	short-chain acylcarnitine	-0.001	0.056
C-8	C08	C-8	long-chain acylcarnitine	-0.046	-0.111
C-8:1	C081	C-8:1	long-chain acylcarnitine	0.208	0.333
C-10	C10	C-10	long-chain acylcarnitine	0.123	0.086
C-10:1	C101	C-10:1	long-chain acylcarnitine	0.066	0.131
C-12	C12	C-12	long-chain acylcarnitine	0.198	0.204
C-12:1	C121	C-12:1	long-chain acylcarnitine	0.046	0.101
C-14	C14	C-14	long-chain acylcarnitine	0.240	0.206
C14:1	C141	C14:1	long-chain acylcarnitine	0.045	-0.026
C-14OH	C14OH	C-14OH	3-hydroxy long-chain acylcarnitine	-0.023	0.063
C-16	C16	C-16	long-chain acylcarnitine	0.377	0.368
C-16OH	C16OH	C-16OH	3-hydroxy long-chain acylcarnitine	-0.070	0.021
C-18	C18	C-18	long-chain acylcarnitine	0.198	0.162
C-18:1	C181	C-18:1	long-chain acylcarnitine	0.313	0.221
C-18:1OH	C181OH	C-18:1OH	3-hydroxy long-chain acylcarnitine	-0.019	0.016
C-18:2	C182	C-18:2	long-chain acylcarnitine	0.296	0.106
C-18OH	C18OH	C-18OH	3-hydroxy long-chain acylcarnitine	-0.031	-0.031
CIT	CIT	Citrulline	amino acid	-0.127	-0.001
FC	FC	Free Carnitine	free carnitine	0.072	0.191
GLY	GLY	Glycine	amino acid	0.151	0.308
MET	MET	Methionine	amino acid	0.070	0.261

ORN	ORN	Ornithine	amino acid	0.220	0.229
OXP	OXP	5-Oxoproline	amino acid	0.056	-
PHE	PHE	Phenylalanine	amino acid	0.093	0.148
PRO	PRO	Proline	amino acid	0.046	-
C-14:1/C-12:1	R14_2	C14:1 / C12:1 Ratio	long-chain acylcarnitine	-0.019	-0.079
C-3/C-2	R3_2	C03 / C02 Ratio	short-chain acylcarnitine	-0.098	-0.071
C-8/C-10	R8_10	C08 / C10 Ratio	short-chain acylcarnitine	-0.152	-0.177
ARG/ORN	RA_O	Arginine/Ornithine Ratio	amino acid	-0.058	-
CIT/ARG	RC_A	Citrulline/Arginine Ratio	amino acid	-0.165	-0.207
FC/(C-16 + C-18:1)	RF_C	FC / (C16 + C18:1) Ratio	free carnitine	-0.227	-0.115
PHE/TYR	RF_Y	Phenylalanine/Tyrosine Ratio	amino acid	0.160	0.024
LEU/ALA	RL_A	Leucine/Alanine Ratio	amino acid	-0.100	-0.241
ORN/CIT	RO_C	Ornithine/Citrulline Ratio	amino acid	0.340	0.271
TYR	TYR	Tyrosine	amino acid	-0.107	0.032
VAL	VAL	Valine	amino acid	0.014	0.097
XLE	XLE	Leucine/Isoleucine	amino acid	0.069	0.123

Table S6. Subgroup discovery results for the top 25 subgroups in each outcome.

Subgroup Definition	% of Dataset	Subgroup Size	Subgroup Rank	Cumulative AUPRC	Subgroup AUPRC	Cumulative AUROC	Subgroup AUROC	Outcome	Dataset Split
C03DCrc_q-5==3 AND C051rc_q-2==0 AND C05rc_q-5==0 AND XLErc_q-3==0	1.30%	68	1	0.998	0.998	0.994	0.994	BPD	K-Fold Test
C05rc_q-3==0 AND C182rc_q-2==0 AND RAOrc_q-5==0 AND XLErc_q-5==1	2.86%	88	2	0.993	0.991	0.983	0.977	BPD	K-Fold Test
C03DCrc_q-5==3 AND C05rc_q-2==0 AND OXPrc_q-2==1 AND PHErc_q-3==0	3.70%	72	3	0.991	0.993	0.980	0.987	BPD	K-Fold Test
C03DCrc_q-5==3 AND C05rc_q-2==0 AND C12rc_q-5==4 AND OXPrc_q-2==1	4.16%	71	4	0.990	0.997	0.979	0.988	BPD	K-Fold Test
C03DCrc_q-5==3 AND C051rc_q-2==0 AND C05rc_q-3==0 AND C12rc_q-5==4	4.30%	57	5	0.989	1.000	0.978	1.000	BPD	K-Fold Test
C03DCrc_q-5==3 AND C05rc_q-5==0 AND RFCrc_q-3==0 AND XLErc_q-3==0	4.39%	66	6	0.988	0.999	0.975	0.991	BPD	K-Fold Test
C03DCrc_q-5==3 AND C051rc_q-3==0 AND C05rc_q-3==0 AND VALrc_q-3==0	4.74%	72	7	0.988	0.992	0.976	0.986	BPD	K-Fold Test
C03DCrc_q-5==3 AND C05rc_q-3==0 AND RFCrc_q-3==0 AND XLErc_q-3==0	4.80%	78	8	0.986	0.996	0.971	0.981	BPD	K-Fold Test
ORNrc_q-5==1 AND PROrc_q-3==1 AND RCArc_q-5==4 AND RLArc_q-2==0	5.64%	58	9	0.986	0.999	0.973	0.997	BPD	K-Fold Test
C03DCrc_q-5==3 AND C05rc_q-3==0 AND C12rc_q-5==4 AND OXPrc_q-2==1	5.64%	60	10	0.986	0.999	0.973	0.995	BPD	K-Fold Test
C03DCrc_q-5==3 AND C05rc_q-2==0 AND C081rc_q-3==1 AND C12rc_q-2==1	5.97%	73	11	0.985	0.995	0.972	0.984	BPD	K-Fold Test
ARGrc_q-3==0 AND C03DCrc_q-5==3 AND C05rc_q-3==0 AND RLArc_q-5==0	6.04%	75	12	0.985	0.994	0.971	0.982	BPD	K-Fold Test
C03DCrc_q-5==3 AND C05rc_q-2==0 AND C081rc_q-3==1 AND C12rc_q-3==2	6.04%	59	13	0.985	0.999	0.971	0.995	BPD	K-Fold Test
C05OHrc_q-2==0 AND ORNrc_q-5==1 AND OXPrc_q-5==4 AND PHErc_q-2==0	6.77%	54	14	0.986	1.000	0.974	1.000	BPD	K-Fold Test
C03DCrc_q-5==3 AND C05rc_q-2==0 AND C12rc_q-2==1 AND RLArc_q-3==0	6.91%	107	15	0.983	0.987	0.971	0.961	BPD	K-Fold Test
C03DCrc_q-5==3 AND C051rc_q-2==0 AND C05rc_q-3==0 AND VALrc_q-3==0	6.93%	89	16	0.982	0.987	0.968	0.971	BPD	K-Fold Test
C03DCrc_q-5==3 AND C05rc_q-3==0 AND RCArc_q-3==2 AND RFCrc_q-3==0	7.16%	83	17	0.980	0.996	0.963	0.975	BPD	K-Fold Test
C03DCrc_q-5==3 AND C05rc_q-2==0 AND C12rc_q-3==2 AND FCrc_q-2==0	7.18%	66	18	0.979	0.998	0.962	0.988	BPD	K-Fold Test
C03DCrc_q-5==3 AND C05rc_q-5==0	7.21%	53	19	0.980	1.000	0.963	1.000	BPD	K-Fold Test

AND RAOrc_q-3==0 AND RFCrc_q-5==0									
C05rc_q-5==0 AND CITrc_q-2==1 AND GLYrc_q-2==1 AND RAOrc_q-5==0	7.79%	60	20	0.982	0.999	0.965	0.993	BPD	K-Fold Test
ARGrc_q-3==0 AND C03DCrc_q-5==3 AND C05rc_q-5==0 AND RFCrc_q-3==0	7.79%	72	21	0.982	0.998	0.965	0.982	BPD	K-Fold Test
C03DCrc_q-5==3 AND C051rc_q-2==0 AND C05rc_q-2==0 AND C12rc_q-5==4	7.79%	68	22	0.982	0.997	0.965	0.986	BPD	K-Fold Test
C03DCrc_q-5==3 AND C05rc_q-3==0 AND C12rc_q-2==1 AND RLArc_q-3==0	7.79%	89	23	0.982	0.993	0.965	0.970	BPD	K-Fold Test
C02rc_q-3==1 AND C05rc_q-2==0 AND OXPrc_q-2==1 AND PHErc_q-5==0	8.86%	89	24	0.980	0.985	0.962	0.970	BPD	K-Fold Test
ARGrc_q-3==0 AND C03DCrc_q-5==3 AND C05rc_q-5==0 AND RFCrc_q-5==0	8.86%	59	25	0.980	0.999	0.962	0.993	BPD	K-Fold Test
C03DCrc_q-5==3 AND C051rc_q-2==0 AND C05rc_q-5==0 AND XLErc_q-3==0	1.11%	19	1	0.822	0.822	0.790	0.790	BPD	Holdout Validation
C05rc_q-3==0 AND C182rc_q-2==0 AND RAOrc_q-5==0 AND XLErc_q-5==1	2.46%	26	2	0.723	0.680	0.743	0.708	BPD	Holdout Validation
C03DCrc_q-5==3 AND C05rc_q-2==0 AND OXPrc_q-2==1 AND PHErc_q-3==0	3.16%	16	3	0.770	0.981	0.714	0.733	BPD	Holdout Validation
C03DCrc_q-5==3 AND C05rc_q-2==0 AND C12rc_q-5==4 AND OXPrc_q-2==1	3.51%	17	4	0.779	0.980	0.723	0.905	BPD	Holdout Validation
C03DCrc_q-5==3 AND C051rc_q-2==0 AND C05rc_q-3==0 AND C12rc_q-5==4	3.51%	10	5	0.779	0.985	0.723	0.938	BPD	Holdout Validation
C03DCrc_q-5==3 AND C05rc_q-5==0 AND RFCrc_q-3==0 AND XLErc_q-3==0	3.57%	16	6	0.776	0.873	0.725	0.836	BPD	Holdout Validation
C03DCrc_q-5==3 AND C051rc_q-3==0 AND C05rc_q-3==0 AND VALrc_q-3==0	3.74%	18	7	0.782	0.783	0.732	0.688	BPD	Holdout Validation
C03DCrc_q-5==3 AND C05rc_q-3==0 AND RFCrc_q-3==0 AND XLErc_q-3==0	3.74%	18	8	0.782	0.868	0.732	0.785	BPD	Holdout Validation
ORNrc_q-5==1 AND PROrc_q-3==1 AND RCArc_q-5==4 AND RLArc_q-2==0	4.15%	14	9	0.797	0.990	0.752	0.975	BPD	Holdout Validation
C03DCrc_q-5==3 AND C05rc_q-3==0 AND C12rc_q-5==4 AND OXPrc_q-2==1	4.15%	13	10	0.797	0.992	0.752	0.955	BPD	Holdout Validation
C03DCrc_q-5==3 AND C05rc_q-2==0 AND C081rc_q-3==1 AND C12rc_q-2==1	4.56%	16	11	0.804	0.993	0.766	0.979	BPD	Holdout Validation
ARGrc_q-3==0 AND C03DCrc_q-5==3 AND C05rc_q-3==0 AND RLArc_q-5==0	4.62%	14	12	0.803	0.807	0.771	0.700	BPD	Holdout Validation
C03DCrc_q-5==3 AND C05rc_q-2==0 AND C081rc_q-3==1 AND C12rc_q-3==2	4.62%	11	13	0.803	0.990	0.771	0.900	BPD	Holdout Validation

C05OHrc_q-2==0 AND ORNrc_q-5==1 AND OXPrc_q-5==4 AND PHErc_q-2==0	5.79%	25	14	0.800	0.849	0.790	0.857	BPD	Holdout Validation
C03DCrc_q-5==3 AND C05rc_q-2==0 AND C12rc_q-2==1 AND RLArc_q-3==0	6.03%	25	15	0.799	0.979	0.797	0.939	BPD	Holdout Validation
C03DCrc_q-5==3 AND C051rc_q-2==0 AND C05rc_q-3==0 AND VALrc_q-3==0	6.03%	23	16	0.799	0.827	0.797	0.738	BPD	Holdout Validation
C03DCrc_q-5==3 AND C05rc_q-3==0 AND RCArc_q-3==2 AND RFCrc_q-3==0	6.26%	23	17	0.807	0.884	0.803	0.814	BPD	Holdout Validation
C03DCrc_q-5==3 AND C05rc_q-2==0 AND C12rc_q-3==2 AND FCrc_q-2==0	6.26%	10	18	0.807	1.000	0.803	1.000	BPD	Holdout Validation
C03DCrc_q-5==3 AND C05rc_q-5==0 AND RAOrc_q-3==0 AND RFCrc_q-5==0	6.26%	13	19	0.807	0.869	0.803	0.733	BPD	Holdout Validation
C05rc_q-5==0 AND CITrc_q-2==1 AND GLYrc_q-2==1 AND RAOrc_q-5==0	6.50%	13	20	0.791	0.859	0.789	0.600	BPD	Holdout Validation
ARGrc_q-3==0 AND C03DCrc_q-5==3 AND C05rc_q-5==0 AND RFCrc_q-3==0	6.50%	15	21	0.791	0.873	0.789	0.795	BPD	Holdout Validation
C03DCrc_q-5==3 AND C051rc_q-2==0 AND C05rc_q-2==0 AND C12rc_q-5==4	6.50%	11	22	0.791	0.985	0.789	0.958	BPD	Holdout Validation
C03DCrc_q-5==3 AND C05rc_q-3==0 AND C12rc_q-2==1 AND RLArc_q-3==0	6.50%	19	23	0.791	0.991	0.789	0.967	BPD	Holdout Validation
C02rc_q-3==1 AND C05rc_q-2==0 AND OXPrc_q-2==1 AND PHErc_q-5==0	7.78%	28	24	0.814	0.888	0.796	0.719	BPD	Holdout Validation
ARGrc_q-3==0 AND C03DCrc_q-5==3 AND C05rc_q-5==0 AND RFCrc_q-5==0	7.78%	14	25	0.814	0.873	0.796	0.727	BPD	Holdout Validation
C181rc_q-3==2 AND OXPrc_q-2==1 AND PHErc_q-5==0 AND RCArc_q-3==2	1.18%	62	1	1.000	1.000	1.000	1.000	IVH	K-Fold Test
OXPrc_q-2==1 AND PHErc_q-5==0 AND RCArc_q-3==2 AND RLArc_q-2==0	3.30%	165	2	0.952	0.953	0.936	0.933	IVH	K-Fold Test
OXPrc_q-2==1 AND PHErc_q-5==0 AND RCArc_q-3==2 AND RLArc_q-3==0	3.30%	138	3	0.952	0.957	0.936	0.940	IVH	K-Fold Test
C02rc_q-2==0 AND OXPrc_q-3==2 AND PHErc_q-5==0 AND RLArc_q-5==0	3.57%	52	4	0.951	0.996	0.935	0.994	IVH	K-Fold Test
C02rc_q-3==1 AND C051rc_q-3==0 AND OXPrc_q-2==1 AND PHErc_q-5==0	4.03%	61	5	0.949	0.993	0.931	0.984	IVH	K-Fold Test
C02rc_q-3==1 AND C051rc_q-2==0 AND OXPrc_q-2==1 AND PHErc_q-5==0	4.03%	61	6	0.949	0.993	0.931	0.984	IVH	K-Fold Test
C181rc_q-3==2 AND OXPrc_q-3==2 AND PHErc_q-5==0 AND RCArc_q-3==2	4.03%	46	7	0.949	1.000	0.931	1.000	IVH	K-Fold Test
C181rc_q-5==4 AND OXPrc_q-2==1 AND	4.18%	46	8	0.953	1.000	0.934	1.000	IVH	K-Fold Test

PHErc_q-5==0 AND RFCrc_q-2==0									
C051rc_q-3==0 AND OXPrc_q-2==1 AND PHErc_q-5==0 AND RLArc_q-2==0	4.64%	143	9	0.943	0.960	0.920	0.935	IVH	K-Fold Test
C051rc_q-2==0 AND OXPrc_q-2==1 AND PHErc_q-5==0 AND RLArc_q-2==0	4.64%	143	10	0.943	0.960	0.920	0.935	IVH	K-Fold Test
ALArc_q-2==1 AND ORNrc_q-5==1 AND RCArc_q-3==2 AND ROCrc_q-5==0	5.55%	51	11	0.937	0.978	0.925	0.994	IVH	K-Fold Test
OXPrc_q-2==1 AND PHErc_q-5==0 AND RFYrc_q-2==0 AND RLArc_q-2==0	6.10%	206	12	0.922	0.940	0.905	0.914	IVH	K-Fold Test
METrc_q-3==0 AND OXPrc_q-5==3 AND PHErc_q-5==0 AND RFYrc_q-2==0	6.28%	72	13	0.921	0.980	0.906	0.971	IVH	K-Fold Test
C181rc_q-3==2 AND OXPrc_q-2==1 AND PHErc_q-5==0 AND RCArc_q-2==1	6.33%	78	14	0.918	0.988	0.904	0.966	IVH	K-Fold Test
OXPrc_q-3==2 AND PHErc_q-5==0 AND RCArc_q-3==2 AND RLArc_q-5==0	6.33%	72	15	0.918	0.986	0.904	0.971	IVH	K-Fold Test
OXPrc_q-2==1 AND PHErc_q-5==0 AND RCArc_q-3==2 AND RLArc_q-5==0	6.33%	106	16	0.918	0.970	0.904	0.949	IVH	K-Fold Test
C182rc_q-3==0 AND CITrc_q-3==1 AND RLArc_q-5==0 AND TYRrc_q-2==0	7.17%	60	17	0.928	0.994	0.908	0.980	IVH	K-Fold Test
C181rc_q-5==4 AND OXPrc_q-2==1 AND PHErc_q-5==0 AND RCArc_q-2==1	7.17%	47	18	0.928	0.999	0.908	0.994	IVH	K-Fold Test
OXPrc_q-3==2 AND PHErc_q-5==0 AND RFYrc_q-2==0 AND RLArc_q-2==0	7.17%	135	19	0.928	0.960	0.908	0.935	IVH	K-Fold Test
ARGrc_q-3==0 AND C181rc_q-5==4 AND OXPrc_q-2==1 AND PHErc_q-5==0	7.17%	42	20	0.928	1.000	0.908	1.000	IVH	K-Fold Test
FCrc_q-2==0 AND OXPrc_q-3==2 AND PHErc_q-5==0 AND RLArc_q-5==0	7.17%	61	21	0.928	0.990	0.908	0.978	IVH	K-Fold Test
OXPrc_q-3==2 AND PHErc_q-2==0 AND RFYrc_q-2==0 AND VALrc_q-5==0	8.05%	122	22	0.922	0.960	0.902	0.939	IVH	K-Fold Test
C03DCrc_q-2==1 AND OXPrc_q-3==2 AND PHErc_q-5==0 AND RFCrc_q-3==0	8.09%	44	23	0.922	0.999	0.902	0.997	IVH	K-Fold Test
OXPrc_q-2==1 AND PHErc_q-5==0 AND ROCrc_q-5==0 AND VALrc_q-5==0	8.16%	72	24	0.922	0.970	0.903	0.968	IVH	K-Fold Test
ORNrc_q-5==1 AND OXPrc_q-2==1 AND RCArc_q-3==2 AND RFYrc_q-2==0	9.31%	139	25	0.915	0.933	0.903	0.931	IVH	K-Fold Test
C181rc_q-3==2 AND OXPrc_q-2==1 AND PHErc_q-5==0 AND RCArc_q-3==2	1.34%	23	1	0.643	0.643	0.667	0.667	IVH	Holdout Validation
OXPrc_q-2==1 AND PHErc_q-5==0 AND RCArc_q-3==2 AND RLArc_q-2==0	3.92%	65	2	0.692	0.685	0.724	0.712	IVH	Holdout Validation

OXPrc_q-2==1 AND PHErc_q-5==0 AND RCArc_q-3==2 AND RLArc_q-3==0	3.92%	53	3	0.692	0.665	0.724	0.730	IVH	Holdout Validation
C02rc_q-2==0 AND OXPrc_q-3==2 AND PHErc_q-5==0 AND RLArc_q-5==0	4.15%	19	4	0.698	0.628	0.719	0.636	IVH	Holdout Validation
C02rc_q-3==1 AND C051rc_q-3==0 AND OXPrc_q-2==1 AND PHErc_q-5==0	4.62%	20	5	0.729	0.764	0.731	0.653	IVH	Holdout Validation
C02rc_q-3==1 AND C051rc_q-2==0 AND OXPrc_q-2==1 AND PHErc_q-5==0	4.79%	25	6	0.717	0.748	0.712	0.570	IVH	Holdout Validation
C181rc_q-3==2 AND OXPrc_q-3==2 AND PHErc_q-5==0 AND RCArc_q-3==2	4.79%	15	7	0.717	0.581	0.712	0.460	IVH	Holdout Validation
C181rc_q-5==4 AND OXPrc_q-2==1 AND PHErc_q-5==0 AND RFCrc_q-2==0	4.91%	20	8	0.718	0.636	0.716	0.604	IVH	Holdout Validation
C051rc_q-3==0 AND OXPrc_q-2==1 AND PHErc_q-5==0 AND RLArc_q-2==0	5.20%	52	9	0.731	0.723	0.735	0.682	IVH	Holdout Validation
C051rc_q-2==0 AND OXPrc_q-2==1 AND PHErc_q-5==0 AND RLArc_q-2==0	5.38%	63	10	0.714	0.685	0.728	0.668	IVH	Holdout Validation
ALArc_q-2==1 AND ORNrc_q-5==1 AND RCArc_q-3==2 AND ROCrc_q-5==0	6.55%	22	11	0.698	0.449	0.725	0.620	IVH	Holdout Validation
OXPrc_q-2==1 AND PHErc_q-5==0 AND RFYrc_q-2==0 AND RLArc_q-2==0	6.66%	63	12	0.697	0.705	0.722	0.725	IVH	Holdout Validation
METrc_q-3==0 AND OXPrc_q-5==3 AND PHErc_q-5==0 AND RFYrc_q-2==0	6.84%	22	13	0.700	0.651	0.722	0.643	IVH	Holdout Validation
C181rc_q-3==2 AND OXPrc_q-2==1 AND PHErc_q-5==0 AND RCArc_q-2==1	6.95%	31	14	0.696	0.665	0.715	0.667	IVH	Holdout Validation
OXPrc_q-3==2 AND PHErc_q-5==0 AND RCArc_q-3==2 AND RLArc_q-5==0	6.95%	29	15	0.696	0.611	0.715	0.591	IVH	Holdout Validation
OXPrc_q-2==1 AND PHErc_q-5==0 AND RCArc_q-3==2 AND RLArc_q-5==0	6.95%	44	16	0.696	0.624	0.715	0.708	IVH	Holdout Validation
C182rc_q-3==0 AND CITrc_q-3==1 AND RLArc_q-5==0 AND TYRrc_q-2==0	7.66%	15	17	0.716	0.933	0.727	0.889	IVH	Holdout Validation
C181rc_q-5==4 AND OXPrc_q-2==1 AND PHErc_q-5==0 AND RCArc_q-2==1	7.66%	20	18	0.716	0.619	0.727	0.646	IVH	Holdout Validation
OXPrc_q-3==2 AND PHErc_q-5==0 AND RFYrc_q-2==0 AND RLArc_q-2==0	7.66%	43	19	0.716	0.759	0.727	0.762	IVH	Holdout Validation
ARGrc_q-3==0 AND C181rc_q-5==4 AND OXPrc_q-2==1 AND PHErc_q-5==0	7.66%	20	20	0.716	0.619	0.727	0.646	IVH	Holdout Validation
FCrc_q-2==0 AND OXPrc_q-3==2 AND PHErc_q-5==0 AND RLArc_q-5==0	7.66%	24	21	0.716	0.667	0.727	0.707	IVH	Holdout Validation
OXPrc_q-3==2 AND PHErc_q-2==0 AND	8.30%	35	22	0.724	0.932	0.743	0.918	IVH	Holdout Validation

RFYrc_q-2==0 AND VALrc_q-5==0									
C03DCrc_q-2==1 AND OXPrc_q-3==2 AND PHErc_q-5==0 AND RFCrc_q-3==0	8.30%	14	23	0.724	0.528	0.743	0.378	IVH	Holdout Validation
OXPrc_q-2==1 AND PHErc_q-5==0 AND ROCrc_q-5==0 AND VALrc_q-5==0	8.36%	29	24	0.727	0.834	0.744	0.858	IVH	Holdout Validation
ORNrc_q-5==1 AND OXPrc_q-2==1 AND RCArc_q-3==2 AND RFYrc_q-2==0	9.29%	51	25	0.744	0.824	0.761	0.853	IVH	Holdout Validation
C04rc_q-2==0 AND C12rc_q-5==4 AND RFCrc_q-2==0 AND RLArc_q-5==0	3.04%	106	1	1.000	1.000	0.990	0.990	NEC	K-Fold Test
C04rc_q-2==0 AND C05rc_q-3==0 AND C12rc_q-5==4 AND RLArc_q-5==0	3.47%	103	2	0.999	1.000	0.976	0.990	NEC	K-Fold Test
C05rc_q-2==0 AND C14rc_q-3==2 AND OXPrc_q-2==1 AND RCArc_q-3==2	7.16%	176	3	0.996	0.995	0.944	0.939	NEC	K-Fold Test
ARGrc_q-3==0 AND C12rc_q-5==4 AND RFCrc_q-2==0 AND RLArc_q-5==0	7.42%	98	4	0.995	1.000	0.939	0.986	NEC	K-Fold Test
C14rc_q-3==2 AND OXPrc_q-2==1 AND RCArc_q-3==2 AND RFCrc_q-5==0	7.56%	91	5	0.995	1.000	0.938	0.989	NEC	K-Fold Test
C12rc_q-3==2 AND OXPrc_q-2==1 AND RCArc_q-3==2 AND RFCrc_q-5==0	7.96%	84	6	0.995	1.000	0.938	0.994	NEC	K-Fold Test
C181rc_q-2==1 AND C182rc_q-2==0 AND OXPrc_q-2==1 AND RCArc_q-3==2	9.40%	174	7	0.991	0.989	0.930	0.934	NEC	K-Fold Test
ALArc_q-5==3 AND C04rc_q-2==0 AND C05rc_q-3==0 AND RLArc_q-2==0	11.34%	113	8	0.992	0.998	0.933	0.968	NEC	K-Fold Test
C02rc_q-3==1 AND C16rc_q-5==4 AND C182rc_q-2==0 AND RCArc_q-2==1	12.55%	102	9	0.992	0.999	0.934	0.976	NEC	K-Fold Test
C12rc_q-2==1 AND RCArc_q-3==2 AND RFCrc_q-2==0 AND RLArc_q-5==0	13.29%	154	10	0.992	0.997	0.925	0.943	NEC	K-Fold Test
C04rc_q-2==0 AND C05rc_q-5==0 AND C12rc_q-5==4 AND RLArc_q-5==0	13.29%	88	11	0.992	1.000	0.925	0.988	NEC	K-Fold Test
C04rc_q-3==0 AND C051rc_q-5==0 AND OXPrc_q-2==1 AND PHErc_q-3==0	14.47%	85	12	0.992	1.000	0.927	0.991	NEC	K-Fold Test
C04rc_q-3==0 AND C051rc_q-3==0 AND OXPrc_q-2==1 AND PHErc_q-3==0	14.47%	85	13	0.992	1.000	0.927	0.991	NEC	K-Fold Test
ALArc_q-5==3 AND C05rc_q-3==0 AND C16rc_q-3==2	14.95%	82	14	0.992	1.000	0.926	0.994	NEC	K-Fold Test
ALArc_q-5==3 AND C05rc_q-3==0 AND C16rc_q-2==1 AND C16rc_q-3==2	14.95%	82	15	0.992	1.000	0.926	0.994	NEC	K-Fold Test
ALArc_q-2==1 AND ALArc_q-5==3 AND C05rc_q-3==0 AND C16rc_q-3==2	14.95%	82	16	0.992	1.000	0.926	0.994	NEC	K-Fold Test
ALArc_q-5==3 AND C05rc_q-2==0 AND	14.95%	82	17	0.992	1.000	0.926	0.994	NEC	K-Fold Test

C05rc_q-3==0 AND C16rc_q-3==2									
C04rc_q-2==0 AND C05rc_q-5==0 AND C181rc_q-3==2 AND RLArc_q-5==0	15.24%	76	18	0.992	1.000	0.928	1.000	NEC	K-Fold Test
ALArc_q-5==3 AND C04rc_q-2==0 AND C05rc_q-3==0 AND RCArc_q-2==1	15.38%	86	19	0.992	0.999	0.929	0.989	NEC	K-Fold Test
C02rc_q-3==1 AND C121rc_q-2==1 AND C16rc_q-5==4 AND RCArc_q-2==1	15.64%	75	20	0.992	1.000	0.929	1.000	NEC	K-Fold Test
C04rc_q-2==0 AND C05rc_q-3==0 AND RAOrc_q-3==0 AND RLArc_q-5==0	16.47%	178	21	0.987	0.992	0.908	0.928	NEC	K-Fold Test
ALArc_q-5==3 AND C05rc_q-3==0 AND RAOrc_q-2==0 AND RCArc_q-2==1	16.70%	106	22	0.987	0.997	0.907	0.970	NEC	K-Fold Test
ALArc_q-5==3 AND C05rc_q-3==0 AND RAOrc_q-2==0 AND RCArc_q-3==2	16.70%	84	23	0.987	0.999	0.907	0.988	NEC	K-Fold Test
C04rc_q-5==0 AND C14rc_q-3==2 AND OXPrc_q-2==1 AND RCArc_q-3==2	16.76%	79	24	0.987	1.000	0.907	0.994	NEC	K-Fold Test
C051rc_q-5==0 AND C05rc_q-3==0 AND OXPrc_q-2==1 AND PHErc_q-3==0	17.24%	97	25	0.987	0.998	0.909	0.976	NEC	K-Fold Test
C04rc_q-2==0 AND C12rc_q-5==4 AND RFCrc_q-2==0 AND RLArc_q-5==0	2.25%	27	1	0.993	0.993	0.920	0.920	NEC	Holdout Validation
C04rc_q-2==0 AND C05rc_q-3==0 AND C12rc_q-5==4 AND RLArc_q-5==0	3.17%	34	2	0.967	0.977	0.735	0.774	NEC	Holdout Validation
C05rc_q-2==0 AND C14rc_q-3==2 AND OXPrc_q-2==1 AND RCArc_q-3==2	7.01%	60	3	0.893	0.890	0.661	0.680	NEC	Holdout Validation
ARGrc_q-3==0 AND C12rc_q-5==4 AND RFCrc_q-2==0 AND RLArc_q-5==0	7.42%	25	4	0.894	0.996	0.672	0.957	NEC	Holdout Validation
C14rc_q-3==2 AND OXPrc_q-2==1 AND RCArc_q-3==2 AND RFCrc_q-5==0	7.76%	24	5	0.893	0.843	0.694	0.619	NEC	Holdout Validation
C12rc_q-3==2 AND OXPrc_q-2==1 AND RCArc_q-3==2 AND RFCrc_q-5==0	8.01%	22	6	0.892	0.827	0.720	0.722	NEC	Holdout Validation
C181rc_q-2==1 AND C182rc_q-2==0 AND OXPrc_q-2==1 AND RCArc_q-3==2	9.76%	51	7	0.888	0.950	0.773	0.846	NEC	Holdout Validation
ALArc_q-5==3 AND C04rc_q-2==0 AND C05rc_q-3==0 AND RLArc_q-2==0	12.34%	47	8	0.886	0.921	0.728	0.565	NEC	Holdout Validation
C02rc_q-3==1 AND C16rc_q-5==4 AND C182rc_q-2==0 AND RCArc_q-2==1	13.84%	34	9	0.903	1.000	0.751	1.000	NEC	Holdout Validation
C12rc_q-2==1 AND RCArc_q-3==2 AND RFCrc_q-2==0 AND RLArc_q-5==0	14.68%	47	10	0.906	0.906	0.753	0.709	NEC	Holdout Validation
C04rc_q-2==0 AND C05rc_q-5==0 AND C12rc_q-5==4 AND RLArc_q-5==0	14.68%	29	11	0.906	0.985	0.753	0.815	NEC	Holdout Validation

C04rc_q-3==0 AND C051rc_q-5==0 AND OXPrc_q-2==1 AND PHErc_q-3==0	16.43%	41	12	0.911	0.954	0.747	0.752	NEC	Holdout Validation
C04rc_q-3==0 AND C051rc_q-3==0 AND OXPrc_q-2==1 AND PHErc_q-3==0	16.76%	53	13	0.911	0.960	0.747	0.786	NEC	Holdout Validation
ALArc_q-5==3 AND C05rc_q-3==0 AND C16rc_q-3==2	17.60%	31	14	0.916	0.953	0.749	0.667	NEC	Holdout Validation
ALArc_q-5==3 AND C05rc_q-3==0 AND C16rc_q-2==1 AND C16rc_q-3==2	17.60%	31	15	0.916	0.953	0.749	0.667	NEC	Holdout Validation
ALArc_q-2==1 AND ALArc_q-5==3 AND C05rc_q-3==0 AND C16rc_q-3==2	17.60%	31	16	0.916	0.953	0.749	0.667	NEC	Holdout Validation
ALArc_q-5==3 AND C05rc_q-2==0 AND C05rc_q-3==0 AND C16rc_q-3==2	17.60%	31	17	0.916	0.953	0.749	0.667	NEC	Holdout Validation
C04rc_q-2==0 AND C05rc_q-5==0 AND C181rc_q-3==2 AND RLArc_q-5==0	18.02%	23	18	0.908	0.957	0.735	0.690	NEC	Holdout Validation
ALArc_q-5==3 AND C04rc_q-2==0 AND C05rc_q-3==0 AND RCArc_q-2==1	18.27%	40	19	0.908	0.940	0.732	0.583	NEC	Holdout Validation
C02rc_q-3==1 AND C121rc_q-2==1 AND C16rc_q-5==4 AND RCArc_q-2==1	18.27%	22	20	0.908	1.000	0.732	1.000	NEC	Holdout Validation
C04rc_q-2==0 AND C05rc_q-3==0 AND RAOrc_q-3==0 AND RLArc_q-5==0	19.10%	57	21	0.906	0.862	0.723	0.605	NEC	Holdout Validation
ALArc_q-5==3 AND C05rc_q-3==0 AND RAOrc_q-2==0 AND RCArc_q-2==1	19.35%	42	22	0.906	0.944	0.716	0.599	NEC	Holdout Validation
ALArc_q-5==3 AND C05rc_q-3==0 AND RAOrc_q-2==0 AND RCArc_q-3==2	19.35%	27	23	0.906	0.954	0.716	0.667	NEC	Holdout Validation
C04rc_q-5==0 AND C14rc_q-3==2 AND OXPrc_q-2==1 AND RCArc_q-3==2	19.43%	24	24	0.906	0.847	0.717	0.651	NEC	Holdout Validation
C051rc_q-5==0 AND C05rc_q-3==0 AND OXPrc_q-2==1 AND PHErc_q-3==0	19.77%	44	25	0.904	0.930	0.707	0.653	NEC	Holdout Validation
ARGrc_q-3==0 AND ORNrc_q-3==1 AND RFCrc_q-2==0 AND RLArc_q-5==0	1.24%	76	1	0.997	0.997	0.982	0.982	ROP	K-Fold Test
C05rc_q-3==0 AND C12rc_q-5==4 AND RAOrc_q-2==0 AND RFYrc_q-5==0	2.28%	66	2	0.995	0.997	0.977	0.988	ROP	K-Fold Test
C05rc_q-2==0 AND C12rc_q-5==4 AND RAOrc_q-2==0 AND RFYrc_q-5==0	2.41%	74	3	0.994	0.994	0.973	0.977	ROP	K-Fold Test
ALArc_q-2==1 AND ORNrc_q-5==1 AND RCArc_q-3==2 AND ROCrc_q-5==0	3.32%	61	4	0.991	0.976	0.982	0.990	ROP	K-Fold Test
C12rc_q-3==2 AND OXPrc_q-2==1 AND RCArc_q-3==2 AND RFYrc_q-5==0	4.17%	84	5	0.981	0.968	0.973	0.965	ROP	K-Fold Test
ALArc_q-5==4 AND RAOrc_q-3==0 AND RFCrc_q-2==0 AND ROCrc_q-2==0	4.86%	68	6	0.979	0.983	0.972	0.980	ROP	K-Fold Test

C051rc_q-2==0 AND ORNrc_q-5==1 AND RAOrc_q-3==0 AND RFYrc_q-3==0	5.97%	104	7	0.969	0.961	0.962	0.949	ROP	K-Fold Test
ARGrc_q-2==0 AND C12rc_q-3==2 AND OXPrc_q-2==1 AND RFYrc_q-5==0	6.20%	106	8	0.965	0.965	0.957	0.947	ROP	K-Fold Test
R810rc_q-2==0 AND RAOrc_q-3==0 AND RFYrc_q-2==0 AND XLErc_q-5==1	7.55%	131	9	0.953	0.950	0.945	0.932	ROP	K-Fold Test
ALArc_q-2==1 AND C03DCrc_q-2==1 AND RCArc_q-3==2 AND RFCrc_q-2==0	9.94%	256	10	0.907	0.872	0.913	0.887	ROP	K-Fold Test
C12rc_q-3==2 AND C14rc_q-3==2 AND OXPrc_q-2==1 AND RFYrc_q-5==0	10.84%	125	11	0.903	0.946	0.910	0.934	ROP	K-Fold Test
ALArc_q-2==1 AND C16rc_q-2==1 AND RCArc_q-3==2 AND RFCrc_q-2==0	12.10%	344	12	0.884	0.881	0.894	0.867	ROP	K-Fold Test
GLYrc_q-2==1 AND ORNrc_q-5==1 AND RCArc_q-3==2 AND RLArc_q-5==0	12.54%	102	13	0.879	0.958	0.891	0.947	ROP	K-Fold Test
ALArc_q-3==2 AND ARGrc_q-3==0 AND OXPrc_q-2==1 AND RFCrc_q-2==0	12.80%	178	14	0.881	0.942	0.891	0.909	ROP	K-Fold Test
ARGrc_q-2==0 AND C12rc_q-5==4 AND R810rc_q-2==0 AND RFYrc_q-5==0	13.00%	90	15	0.879	0.974	0.889	0.955	ROP	K-Fold Test
GLYrc_q-2==1 AND ORNrc_q-5==1 AND RCArc_q-3==2 AND TYRrc_q-3==2	13.13%	80	16	0.878	0.921	0.888	0.963	ROP	K-Fold Test
C101rc_q-2==0 AND C10rc_q-5==4 AND C16rc_q-2==1 AND RFYrc_q-5==0	13.37%	49	17	0.877	0.999	0.889	0.998	ROP	K-Fold Test
ALArc_q-2==1 AND GLYrc_q-2==1 AND ORNrc_q-5==1 AND RCArc_q-3==2	13.47%	111	18	0.877	0.933	0.889	0.940	ROP	K-Fold Test
ALArc_q-3==2 AND C05rc_q-3==0 AND RAOrc_q-3==0 AND XLErc_q-5==1	13.52%	55	19	0.877	0.998	0.889	0.989	ROP	K-Fold Test
ALArc_q-5==4 AND PHErc_q-2==0 AND RCArc_q-3==2 AND RFCrc_q-2==0	13.55%	57	20	0.876	0.994	0.889	0.987	ROP	K-Fold Test
ORNrc_q-5==1 AND RAOrc_q-3==0 AND RFYrc_q-3==0 AND TYRrc_q-2==1	13.80%	140	21	0.873	0.921	0.886	0.924	ROP	K-Fold Test
ARGrc_q-3==0 AND ORNrc_q-5==1 AND RFYrc_q-2==0 AND RFYrc_q-3==0	14.04%	176	22	0.866	0.902	0.881	0.909	ROP	K-Fold Test
ARGrc_q-3==0 AND ORNrc_q-5==1 AND RAOrc_q-2==0 AND RFYrc_q-3==0	14.04%	176	23	0.866	0.902	0.881	0.909	ROP	K-Fold Test
C03DCrc_q-2==1 AND C051rc_q-5==1 AND C081rc_q-2==0 AND RCArc_q-3==2	14.95%	112	24	0.863	0.866	0.884	0.939	ROP	K-Fold Test
C03DCrc_q-2==1 AND RCArc_q-3==2 AND RFCrc_q-2==0 AND RLArc_q-2==0	16.17%	344	25	0.846	0.866	0.870	0.865	ROP	K-Fold Test
ARGrc_q-3==0 AND ORNrc_q-	1.17%	24	1	0.729	0.729	0.700	0.700	ROP	Holdout Validation

3==1 AND RFCrc_q-2==0 AND RLArc_q-5==0									
C05rc_q-3==0 AND C12rc_q-5==4 AND RAOrc_q-2==0 AND RFYrc_q-5==0	2.33%	26	2	0.731	0.814	0.684	0.725	ROP	Holdout Validation
C05rc_q-2==0 AND C12rc_q-5==4 AND RAOrc_q-2==0 AND RFYrc_q-5==0	2.57%	31	3	0.745	0.828	0.713	0.783	ROP	Holdout Validation
ALArc_q-2==1 AND ORNrc_q-5==1 AND RCArc_q-3==2 AND ROCrc_q-5==0	3.30%	18	4	0.739	0.647	0.709	0.663	ROP	Holdout Validation
C12rc_q-3==2 AND OXPrc_q-2==1 AND RCArc_q-3==2 AND RFYrc_q-5==0	3.88%	28	5	0.738	0.757	0.783	0.917	ROP	Holdout Validation
ALArc_q-5==4 AND RAOrc_q-3==0 AND RFCrc_q-2==0 AND ROCrc_q-2==0	4.47%	15	6	0.759	0.859	0.771	0.750	ROP	Holdout Validation
C051rc_q-2==0 AND ORNrc_q-5==1 AND RAOrc_q-3==0 AND RFYrc_q-3==0	5.39%	30	7	0.782	0.956	0.791	0.889	ROP	Holdout Validation
ARGrc_q-2==0 AND C12rc_q-3==2 AND OXPrc_q-2==1 AND RFYrc_q-5==0	5.63%	33	8	0.782	0.792	0.788	0.870	ROP	Holdout Validation
R810rc_q-2==0 AND RAOrc_q-3==0 AND RFYrc_q-2==0 AND XLErc_q-5==1	6.99%	47	9	0.796	0.839	0.796	0.841	ROP	Holdout Validation
ALArc_q-2==1 AND C03DCrc_q-2==1 AND RCArc_q-3==2 AND RFCrc_q-2==0	9.37%	80	10	0.800	0.742	0.786	0.725	ROP	Holdout Validation
C12rc_q-3==2 AND C14rc_q-3==2 AND OXPrc_q-2==1 AND RFYrc_q-5==0	10.24%	37	11	0.791	0.679	0.784	0.806	ROP	Holdout Validation
ALArc_q-2==1 AND C16rc_q-2==1 AND RCArc_q-3==2 AND RFCrc_q-2==0	11.46%	106	12	0.774	0.789	0.764	0.705	ROP	Holdout Validation
GLYrc_q-2==1 AND ORNrc_q-5==1 AND RCArc_q-3==2 AND RLArc_q-5==0	11.80%	24	13	0.773	0.843	0.765	0.770	ROP	Holdout Validation
ALArc_q-3==2 AND ARGrc_q-3==0 AND OXPrc_q-2==1 AND RFCrc_q-2==0	11.99%	40	14	0.770	0.594	0.760	0.634	ROP	Holdout Validation
ARGrc_q-2==0 AND C12rc_q-5==4 AND R810rc_q-2==0 AND RFYrc_q-5==0	12.09%	32	15	0.770	0.831	0.762	0.816	ROP	Holdout Validation
GLYrc_q-2==1 AND ORNrc_q-5==1 AND RCArc_q-3==2 AND TYRrc_q-3==2	12.18%	25	16	0.770	0.705	0.765	0.693	ROP	Holdout Validation
C101rc_q-2==0 AND C10rc_q-5==4 AND C16rc_q-2==1 AND RFYrc_q-5==0	12.33%	13	17	0.773	0.983	0.769	0.933	ROP	Holdout Validation
ALArc_q-2==1 AND GLYrc_q-2==1 AND ORNrc_q-5==1 AND RCArc_q-3==2	12.43%	34	18	0.765	0.771	0.765	0.729	ROP	Holdout Validation
ALArc_q-3==2 AND C05rc_q-3==0 AND RAOrc_q-3==0 AND XLErc_q-5==1	12.52%	19	19	0.767	0.745	0.768	0.657	ROP	Holdout Validation
ALArc_q-5==4 AND PHErc_q-2==0 AND	12.52%	14	20	0.767	0.812	0.768	0.515	ROP	Holdout Validation

RCArc_q-3==2 AND RFCrc_q-2==0									
ORNrc_q-5==1 AND RAOrc_q-3==0 AND RFYrc_q-3==0 AND TYRrc_q-2==1	12.86%	42	21	0.765	0.860	0.767	0.851	ROP	Holdout Validation
ARGrc_q-3==0 AND ORNrc_q-5==1 AND RFYrc_q-2==0 AND RFYrc_q-3==0	13.20%	57	22	0.764	0.882	0.765	0.851	ROP	Holdout Validation
ARGrc_q-3==0 AND ORNrc_q-5==1 AND RAOrc_q-2==0 AND RFYrc_q-3==0	13.20%	57	23	0.764	0.882	0.765	0.851	ROP	Holdout Validation
C03DCrc_q-2==1 AND C051rc_q-5==1 AND C081rc_q-2==0 AND RCArc_q-3==2	14.22%	28	24	0.760	0.797	0.775	0.847	ROP	Holdout Validation
C03DCrc_q-2==1 AND RCArc_q-3==2 AND RFCrc_q-2==0 AND RLArc_q-2==0	15.73%	107	25	0.749	0.730	0.767	0.747	ROP	Holdout Validation

Table S7. Comparison of the metabolic health index to standard machine learning approaches using clinical comparator risk variables.

Outcome	Model	Feature Set	mean AUROC	SD AUROC	mean AUPRC	SD AUPRC
BPD	EN	Birthweight, Gestational Age, Infant Sex, Apgar	0.788	0.001	0.771	0.002
BPD	EN	Apgar Scores	0.626	0.002	0.575	0.004
BPD	EN	Birthweight, Gestational Age	0.783	0.002	0.759	0.003
BPD	EN	Birthweight, Gestational Age, Infant Sex	0.787	0.000	0.765	0.001
BPD	Lasso	Birthweight, Gestational Age, Infant Sex, Apgar	0.789	0.000	0.773	0.000
BPD	Lasso	Apgar Scores	0.626	0.001	0.576	0.004
BPD	Lasso	Birthweight, Gestational Age	0.784	0.001	0.760	0.001
BPD	Lasso	Birthweight, Gestational Age, Infant Sex	0.787	0.000	0.765	0.000
BPD	Logistic Regression	Birthweight, Gestational Age, Infant Sex, Apgar	0.789	0.000	0.773	0.001
BPD	Logistic Regression	Apgar Scores	0.626	0.002	0.575	0.003
BPD	Logistic Regression	Birthweight, Gestational Age	0.784	0.000	0.761	0.000
BPD	Logistic Regression	Birthweight, Gestational Age, Infant Sex	0.787	0.000	0.766	0.000
BPD	Deep Learning	NBS Metabolites	0.698	0.004	0.713	0.003
**BPD**	**Metabolic Health Index**	**NBS Metabolites**	**0.843**	**0.006**	**0.879**	**0.004**
IVH	EN	Birthweight, Gestational Age, Infant Sex, Apgar	0.748	0.001	0.695	0.001
IVH	EN	Apgar Scores	0.642	0.001	0.587	0.004
IVH	EN	Birthweight, Gestational Age	0.740	0.002	0.682	0.003
IVH	EN	Birthweight, Gestational Age, Infant Sex	0.742	0.000	0.685	0.001
IVH	Lasso	Birthweight, Gestational Age, Infant Sex, Apgar	0.747	0.000	0.694	0.001
IVH	Lasso	Apgar Scores	0.644	0.001	0.588	0.003
IVH	Lasso	Birthweight, Gestational Age	0.740	0.001	0.683	0.001
IVH	Lasso	Birthweight, Gestational Age, Infant Sex	0.742	0.000	0.685	0.001
IVH	Logistic Regression	Birthweight, Gestational Age, Infant Sex, Apgar	0.747	0.000	0.695	0.000
IVH	Logistic Regression	Apgar Scores	0.643	0.002	0.586	0.004
IVH	Logistic Regression	Birthweight, Gestational Age	0.740	0.000	0.684	0.000
IVH	Logistic Regression	Birthweight, Gestational Age, Infant Sex	0.742	0.000	0.685	0.000
IVH	Deep Learning	NBS Metabolites	0.688	0.003	0.692	0.004
**IVH**	**Metabolic Health Index**	**NBS Metabolites**	**0.836**	**0.006**	**0.869**	**0.004**
NEC	EN	Birthweight, Gestational Age, Infant Sex, Apgar	0.756	0.001	0.876	0.001
NEC	EN	Apgar Scores	0.602	0.003	0.781	0.002
NEC	EN	Birthweight, Gestational Age	0.753	0.002	0.873	0.002
NEC	EN	Birthweight, Gestational Age, Infant Sex	0.757	0.000	0.877	0.000
NEC	Lasso	Birthweight, Gestational Age, Infant Sex, Apgar	0.756	0.000	0.877	0.000
NEC	Lasso	Apgar Scores	0.603	0.002	0.781	0.004

NEC	Lasso	Birthweight, Gestational Age	0.754	0.001	0.875	0.001
NEC	Lasso	Birthweight, Gestational Age, Infant Sex	0.757	0.000	0.876	0.000
NEC	Logistic Regression	Birthweight, Gestational Age, Infant Sex, Apgar	0.756	0.000	0.876	0.000
NEC	Logistic Regression	Apgar Scores	0.604	0.003	0.783	0.003
NEC	Logistic Regression	Birthweight, Gestational Age	0.755	0.000	0.875	0.000
NEC	Logistic Regression	Birthweight, Gestational Age, Infant Sex	0.757	0.000	0.876	0.000
NEC	Deep Learning	NBS Metabolites	0.670	0.003	0.860	0.002
**NEC**	**Metabolic Health Index**	**NBS Metabolites**	**0.734**	**0.007**	**0.938**	**0.003**
ROP	EN	Birthweight, Gestational Age, Infant Sex, Apgar	0.753	0.001	0.659	0.002
ROP	EN	Apgar Scores	0.594	0.002	0.471	0.004
ROP	EN	Birthweight, Gestational Age	0.753	0.002	0.657	0.003
ROP	EN	Birthweight, Gestational Age, Infant Sex	0.754	0.000	0.660	0.001
ROP	Lasso	Birthweight, Gestational Age, Infant Sex, Apgar	0.754	0.000	0.661	0.001
ROP	Lasso	Apgar Scores	0.595	0.001	0.474	0.003
ROP	Lasso	Birthweight, Gestational Age	0.754	0.001	0.658	0.002
ROP	Lasso	Birthweight, Gestational Age, Infant Sex	0.754	0.000	0.660	0.000
ROP	Logistic Regression	Birthweight, Gestational Age, Infant Sex, Apgar	0.754	0.000	0.661	0.001
ROP	Logistic Regression	Apgar Scores	0.595	0.002	0.473	0.004
ROP	Logistic Regression	Birthweight, Gestational Age	0.754	0.000	0.660	0.000
ROP	Logistic Regression	Birthweight, Gestational Age, Infant Sex	0.754	0.000	0.660	0.000
ROP	Deep Learning	NBS Metabolites	0.673	0.004	0.621	0.005
**ROP**	**Metabolic Health Index**	**NBS Metabolites**	**0.815**	**0.008**	**0.807**	**0.008**

Table S8. Integration of clinical features into the metabolic health index.

Outcome	Model	Feature Set	mean AUROC	SD AUROC	mean AUPRC	SD AUPRC
BPD	Deep Learning	NBS Metabolites, Birthweight, Gestational Age, Infant Sex, Apgar	0.599	0.087	0.601	0.081
BPD	Deep Learning	NBS Metabolites, Apgar Scores	0.708	0.002	0.661	0.004
BPD	Deep Learning	NBS Metabolites, Birthweight, Gestational Age	0.257	0.003	0.327	0.002
BPD	Deep Learning	Birthweight, Gestational Age, Infant Sex, Apgar	0.786	0.001	0.775	0.001
BPD	Deep Learning	NBS Metabolites, Birthweight, Gestational Age, Infant Sex	0.754	0.003	0.738	0.007
BPD	Deep Learning	NBS Metabolites Only	0.698	0.004	0.713	0.003
**BPD**	**Metabolic Health Index**	**NBS Metabolites Only**	**0.843**	**0.006**	**0.879**	**0.004**
IVH	Deep Learning	NBS Metabolites, Birthweight, Gestational Age, Infant Sex, Apgar	0.565	0.075	0.546	0.065
IVH	Deep Learning	NBS Metabolites, Apgar Scores	0.700	0.003	0.644	0.003
IVH	Deep Learning	NBS Metabolites, Birthweight, Gestational Age	0.310	0.004	0.346	0.002
IVH	Deep Learning	Birthweight, Gestational Age, Infant Sex, Apgar	0.741	0.001	0.703	0.001
IVH	Deep Learning	NBS Metabolites, Birthweight, Gestational Age, Infant Sex	0.713	0.002	0.671	0.006
IVH	Deep Learning	NBS Metabolites Only	0.688	0.003	0.692	0.004
**IVH**	**Metabolic Health Index**	**NBS Metabolites Only**	**0.836**	**0.006**	**0.869**	**0.004**
NEC	Deep Learning	NBS Metabolites, Birthweight, Gestational Age, Infant Sex, Apgar	0.582	0.075	0.789	0.047
NEC	Deep Learning	NBS Metabolites, Apgar Scores	0.675	0.005	0.825	0.003
NEC	Deep Learning	NBS Metabolites, Birthweight, Gestational Age	0.295	0.006	0.598	0.004
NEC	Deep Learning	Birthweight, Gestational Age, Infant Sex, Apgar	0.755	0.002	0.880	0.001
NEC	Deep Learning	NBS Metabolites, Birthweight, Gestational Age, Infant Sex	0.724	0.003	0.865	0.001
NEC	Deep Learning	NBS Metabolites Only	0.670	0.003	0.860	0.002
**NEC**	**Metabolic Health Index**	**NBS Metabolites Only**	**0.734**	**0.007**	**0.938**	**0.003**
ROP	Deep Learning	NBS Metabolites, Birthweight, Gestational Age, Infant Sex, Apgar	0.589	0.078	0.509	0.073
ROP	Deep Learning	NBS Metabolites, Apgar Scores	0.674	0.002	0.545	0.003
ROP	Deep Learning	NBS Metabolites, Birthweight, Gestational Age	0.283	0.003	0.271	0.001
ROP	Deep Learning	Birthweight, Gestational Age, Infant Sex, Apgar	0.752	0.001	0.662	0.002
ROP	Deep Learning	NBS Metabolites, Birthweight, Gestational Age, Infant Sex	0.728	0.003	0.633	0.007
ROP	Deep Learning	NBS Metabolites Only	0.673	0.004	0.621	0.005
**ROP**	**Metabolic Health Index**	**NBS Metabolites Only**	**0.815**	**0.008**	**0.807**	**0.008**

Table S9. Comparison of demographics and common clinical covariates across adverse outcomes of prematurity.

Outcome	Covariate	Fisher's Exact P Value	Bonferroni-adjusted P Value
BPD	maternal race-ethnicity	0.0454	0.7264
IVH	maternal race-ethnicity	0.0053	0.0848
NEC	maternal race-ethnicity	0.0001	0.0016
ROP	maternal race-ethnicity	0.0083	0.1328
BPD	paternal race-ethnicity	0.1980	1.0
IVH	paternal race-ethnicity	0.1485	1.0
NEC	paternal race-ethnicity	0.0001	0.0016
ROP	paternal race-ethnicity	0.0001	0.0016
BPD	infant sex	0.0056	0.0737
IVH	infant sex	0.0030	0.0478
NEC	infant sex	0.0472	0.7551
ROP	infant sex	2.928×10^{-6}	4.685×10^{-5}
BPD	maternal age	0.3173	1.0
IVH	maternal age	0.0003	0.0048
NEC	maternal age	0.0085	0.1360
ROP	maternal age	0.0266	0.4256

Table S10. Integration of clinical features into the complete metabolic health index pipeline.

Outcome	Model	Metric	Mean	95th CI (Lower)	95th CI (Upper)
BPD	GA-BWT-Sex	AUROC	0.794	0.790	0.797
BPD	GA-BWT-Sex	AUPRC	0.845	0.840	0.848
BPD	GA-BWT-Sex	AUPRC Gain	1.484	1.476	1.490
BPD	NBS Metabolites	AUROC	0.862	0.848	0.872
BPD	NBS Metabolites	AUPRC	0.896	0.887	0.903
**BPD**	**NBS Metabolites**	**AUPRC Gain**	**1.638**	**1.621**	**1.652**
**BPD**	**Covariate Integration**	**AUROC**	**0.873**	**0.864**	**0.882**
**BPD**	**Covariate Integration**	**AUPRC**	**0.907**	**0.901**	**0.913**
BPD	Covariate Integration	AUPRC Gain	1.621	1.609	1.631
IVH	GA-BWT-Sex	AUROC	0.868	0.864	0.871
**IVH**	**GA-BWT-Sex**	**AUPRC**	**0.886**	**0.882**	**0.890**
**IVH**	**GA-BWT-Sex**	**AUPRC Gain**	**1.752**	**1.744**	**1.759**
IVH	NBS Metabolites	AUROC	0.854	0.840	0.865
IVH	NBS Metabolites	AUPRC	0.881	0.875	0.890
IVH	NBS Metabolites	AUPRC Gain	1.714	1.701	1.730
**IVH**	**Covariate Integration**	**AUROC**	**0.869**	**0.859**	**0.877**
IVH	Covariate Integration	AUPRC	0.885	0.877	0.890
IVH	Covariate Integration	AUPRC Gain	1.751	1.735	1.761
NEC	GA-BWT-Sex	AUROC	0.773	0.769	0.775
NEC	GA-BWT-Sex	AUPRC	0.916	0.915	0.918
**NEC**	**GA-BWT-Sex**	**AUPRC Gain**	**1.210**	**1.208**	**1.213**
NEC	NBS Metabolites	AUROC	0.864	0.845	0.880
NEC	NBS Metabolites	AUPRC	0.978	0.974	0.981
NEC	NBS Metabolites	AUPRC Gain	1.125	1.120	1.128
**NEC**	**Covariate Integration**	**AUROC**	**0.889**	**0.876**	**0.908**
**NEC**	**Covariate Integration**	**AUPRC**	**0.980**	**0.977**	**0.984**
NEC	Covariate Integration	AUPRC Gain	1.142	1.138	1.146
ROP	GA-BWT-Sex	AUROC	0.764	0.762	0.767
ROP	GA-BWT-Sex	AUPRC	0.745	0.741	0.750
ROP	GA-BWT-Sex	AUPRC Gain	1.712	1.703	1.722
ROP	NBS Metabolites	AUROC	0.817	0.804	0.826
ROP	NBS Metabolites	AUPRC	0.793	0.781	0.804
**ROP**	**NBS Metabolites**	**AUPRC Gain**	**1.817**	**1.788**	**1.841**
**ROP**	**Covariate Integration**	**AUROC**	**0.832**	**0.815**	**0.846**
**ROP**	**Covariate Integration**	**AUPRC**	**0.878**	**0.866**	**0.885**
ROP	Covariate Integration	AUPRC Gain	1.586	1.564	1.599
